# Supplementary material for: Functional impact of subunit composition and compensation on Drosophila melanogaster nicotinic receptors–targets of neonicotinoids
Source: PLoS Genet. 2023 Feb 16;19(2):e1010522. doi: 10.1371/journal.pgen.1010522 (PMC9934367; doi:10.1371/journal.pgen.1010522)
Supplement: S1 Text — Fig A. Inward current response of oocytes expressing D. melanogaster nAChRs to several concentrations of ACh. Horizontal bar shows application of ACh. Fig B. Effects of α-bungarotoxin (α-BTX) on the response to 100 μM ACh of X. laevis oocytes expressing D. melanogaster nAChRs. (a) Inward currents induced in oocytes expressing D. melanogaster nAChRs in response to 100 μM ACh in the absence and presence of α-BTX. Horizontal lines indicate application of ACh. (b) Bar graph representations of the current amplitude of responses to 100 μM ACh of the nAChR expressing oocytes exposed to 10 nM or 100 nM α-BTX. Error bars are standard error of the mean (n = 5). Fig C. Inward current responses to ACh and neonicotinoids (imidacloprid, thiacloprid, and clothianidin) of oocytes expressing D. melanogaster nAChRs. Horizontal lines indicate application of neonicotinoids. Fig D. t-SNE representations of Dα1, Dα2, Dα3, Dα4, Dβ1, and Dβ2 gene expressions in the adult brain and ventral nerve cord of D. melganogaster. The figure was illustrated by SCope (https://scope.aertslab.org/#/86757313-d473-4f5f-b045-fc035d99451a/*/welcome) using single cell RNA-sequencing data [43]. These six nAChR subunit genes are co-expressed in single cells (See white dots.). Fig E. Inward current responses to ACh and neonicotinoids (imidacloprid, thiacloprid, and clothianidin) of oocytes expressing D. melanogaster Dα2/Dα3/Dα4/Dβ1/Dβ2, Dα2/Dα3/Dβ1/Dβ2/Dβ3, Dα1/Dα3/Dα4/Dβ1/Dβ2, Dα1/Dα3/Dβ1/Dβ2/Dβ3, Dα1/Dα2/Dα4/Dβ1/Dβ2, and Dα1/Dα2/Dβ1/Dβ2/Dβ3 nAChRs. Horizontal lines indicate application of neonicotinoids. Table A. One-way ANOVA of the pEC50 values of ACh for D. melanogaster nAChRs. Table B. One-way ANOVA of the pEC50 values of imidacloprid for D. melanogaster nAChRs. Table C. One-way ANOVA of the Imax values of imidacloprid for D. melanogaster nAChRs. Table D. One-way ANOVA of the pEC50 values of thiacloprid for D. melanogaster nAChRs. Table E. One-way ANOVA of the Imax values of thiacloprid for D. melanogaste [file pgen.1010522.s001.docx]

**Functional impact of subunit composition and compensation on *Drosophila melanogaster* nicotinic receptors – targets of neonicotinoids (Supporting Information)**

Yuma Komori^1,†^, Koichi Takayama^1,†^, Naoki Okamoto^2,†^, Masaki Kamiya^1^, Wataru Koizumi^1^, Makoto Ihara^1^, Daitaro Misawa^3^, Kotaro Kamiya^3^, Yuto Yoshinari^2,‡^, Kazuki Seike^4^, Shu Kondo^5,6^, Hiromu Tanimoto^7^, Ryusuke Niwa^2^, David B. Sattelle^8^ and Kazuhiko Matsuda^1,2,9,^*

^1^Department of Applied Biological Chemistry, Faculty of Agriculture, Kindai University, 3327-204 Nakamachi, Nara 631-8505, Japan

^2^Life Science Center for Survival Dynamics, Tsukuba Advanced Research Alliance (TARA), University of Tsukuba, 1-1-1 Tennodai, Tsukuba, Ibaraki 305-8577, Japan

^3^SyntheticGestalt, KK, 1-6 Naitomachi, Shinjuku, Tokyo, 160-0014, Japan

^4^Degree Programs in Life and Earth Sciences, Graduate School of Science and Technology, University of Tsukuba, Tennodai 1-1-1, Tsukuba, Ibaraki 305-8572, Japan

^5^Department of Biological Science and Technology, Faculty of Advanced Engineering, Tokyo University of Science, Niijuku 6-3-1, Katsushika-ku, Tokyo 125-8585, Japan

^6^Invertebrate Genetics Laboratory, National Institute of Genetics, Yata 111, Mishima, Shizuoka 411-8540, Japan

^7^Graduate School of Life Sciences, Tohoku University, Katahira 2-1-1, Sendai, Miyagi 980-8577, Japan

^8^Centre for Respiratory Biology, Division of Medicine, University College London, Rayne Building, 5 University Street, London WC1E 6JF, UK

^9^Agricultural Technology and Innovation Research Institute, Kindai University, 3327-204 Nakamachi, Nara 631-8505, Japan

^†^These authors equally contributed to this study.

^‡^Current address: Institute for Molecular and Cellular Regulation, Gunma University, 3-39-15 Showa-machi, Maebashi, 371-8512, Japan.

*kmatsuda@nara.kindai.ac.jp


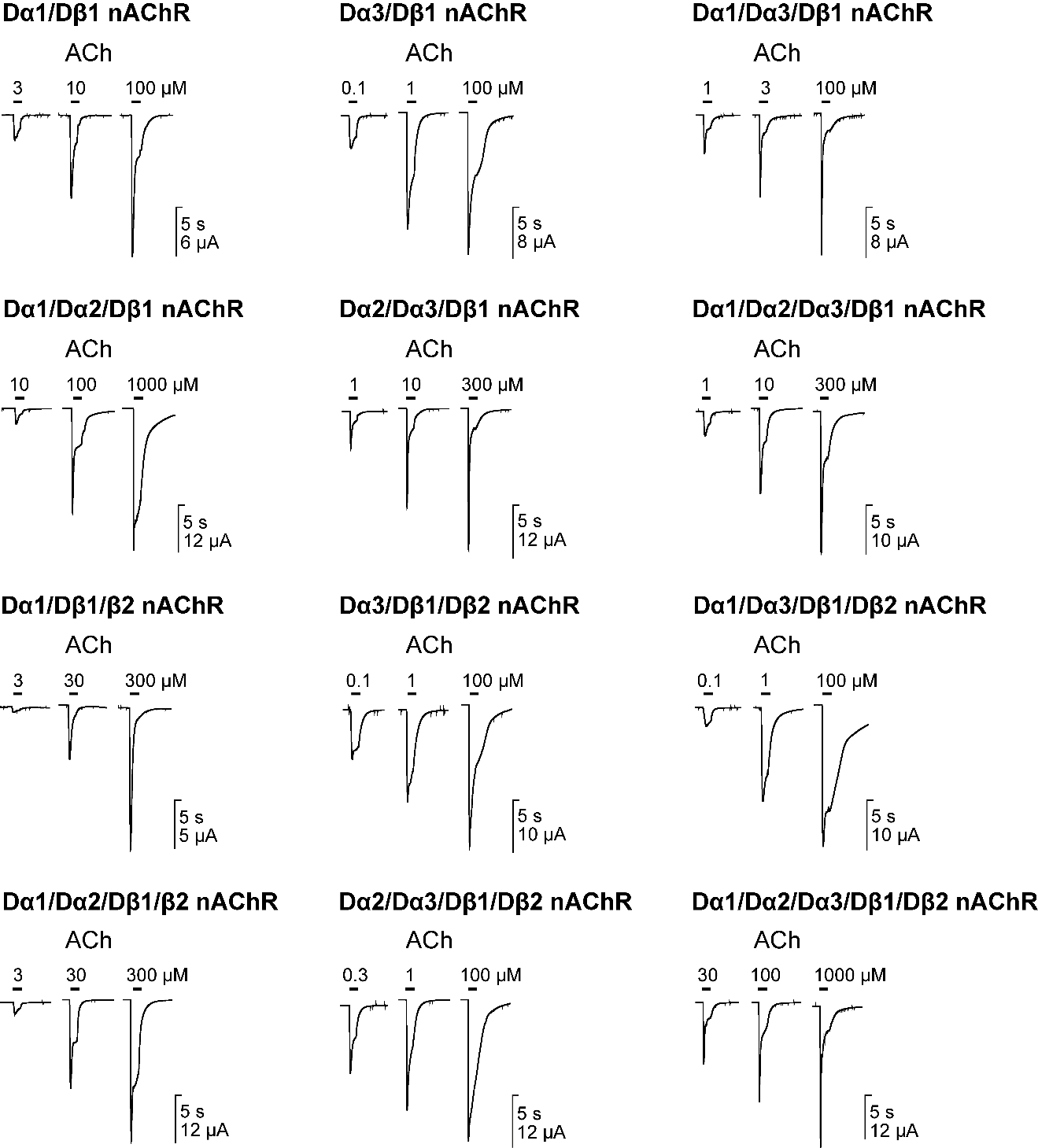


Fig A. Inward current response of oocytes expressing *D. melanogaster* nAChRs to several concentrations of ACh. Horizontal bar shows application of ACh.


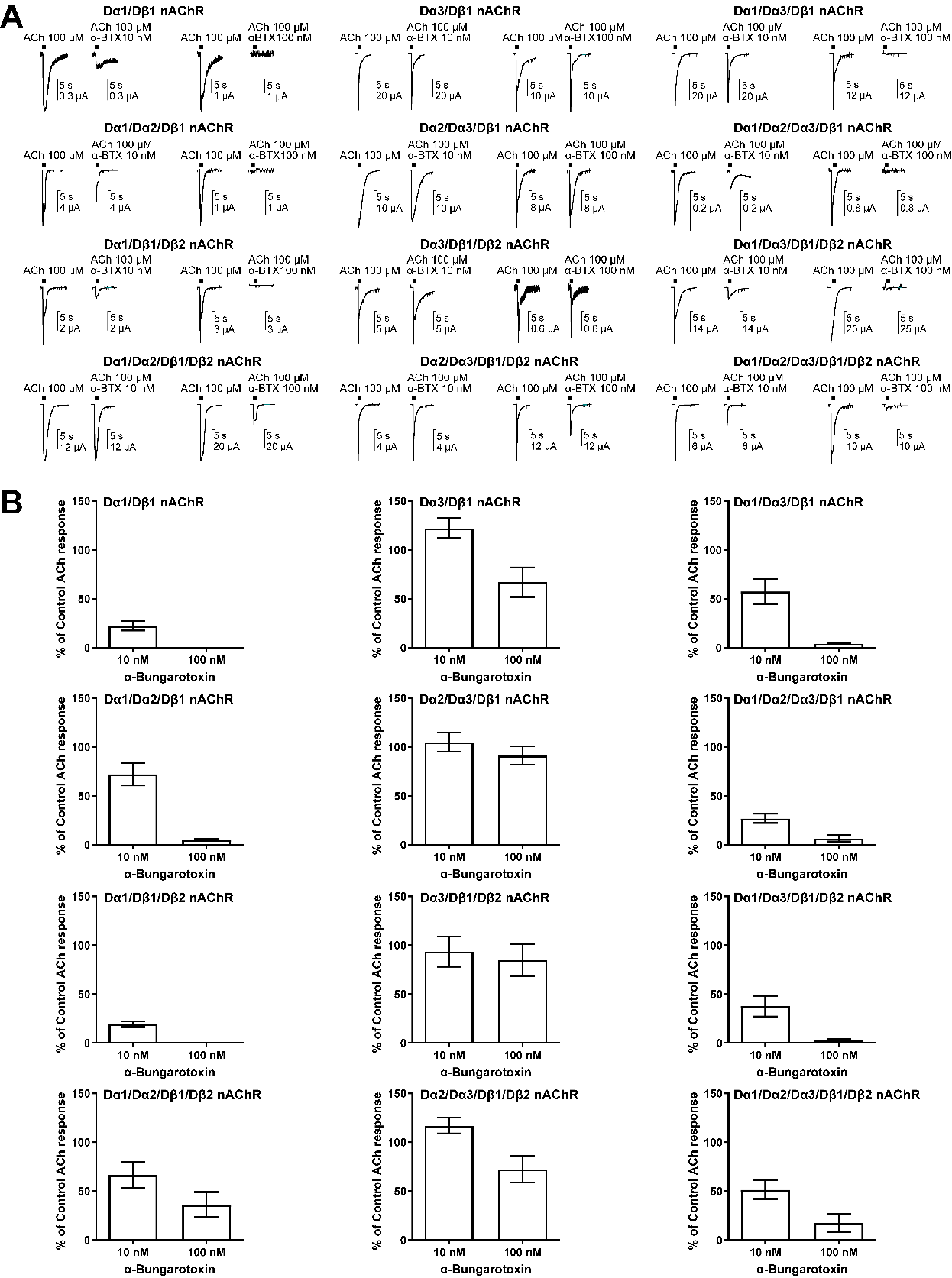


Fig B. Effects of α-bungarotoxin (α-BTX) on the response to 100 µM ACh of *X. laevis* oocytes expressing *D. melanogaster* nAChRs. (A) Inward currents induced in oocytes expressing *D. melanogaster* nAChRs in response to 100 µM ACh in the absence and presence of α-BTX. Horizontal lines indicate application of ACh. (B) Bar graph representations of the current amplitude of responses to 100 µM ACh of the nAChR expressing oocytes exposed to 10 nM or 100 nM α-BTX. Error bars are standard error of the mean (n = 5).


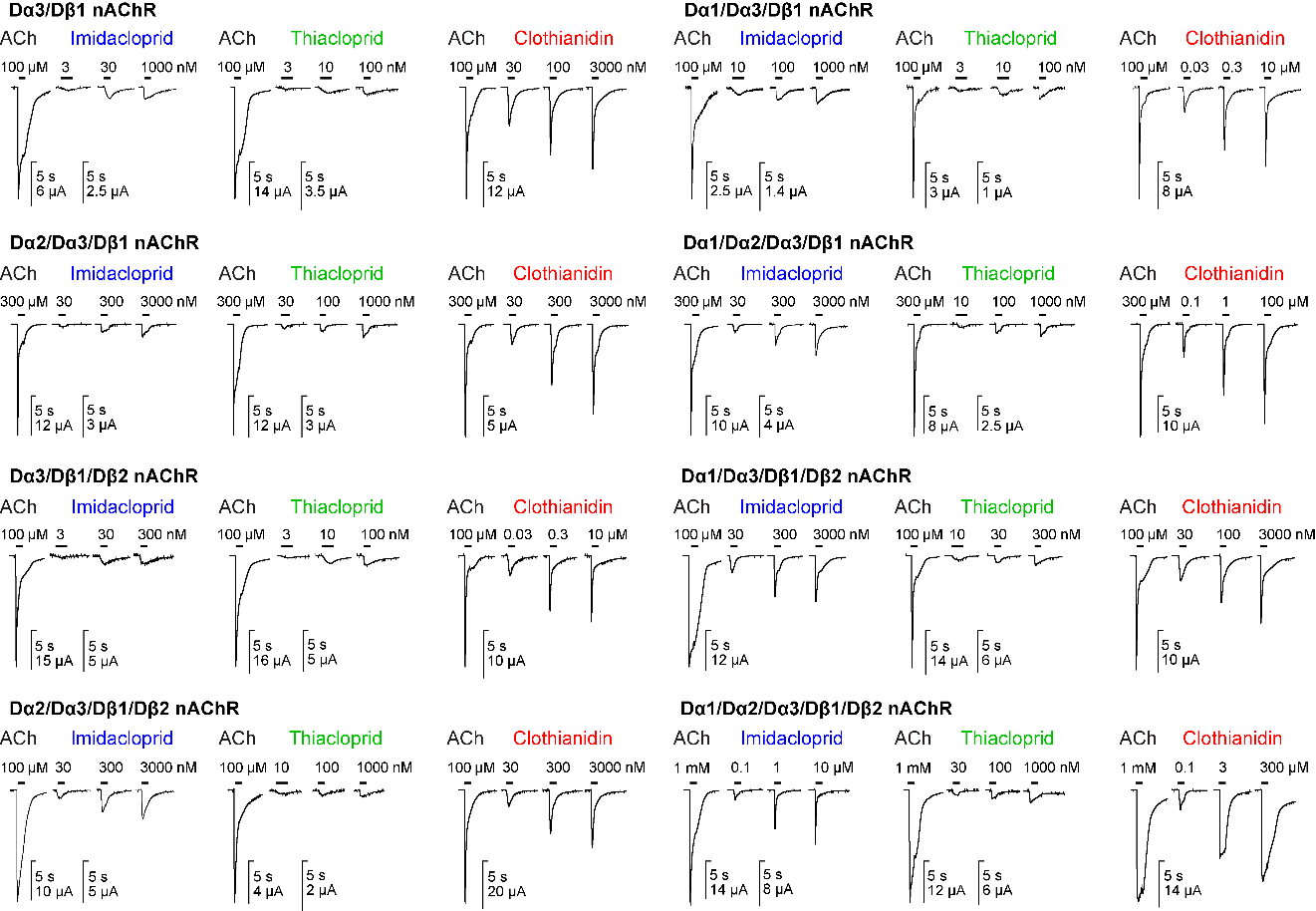


Fig C. Inward current responses to neonicotinoids (imidacloprid, thiacloprid, and clothianidin) of oocytes expressing *D. melanogaster* nAChRs. Horizontal lines indicate application of neonicotinoids.


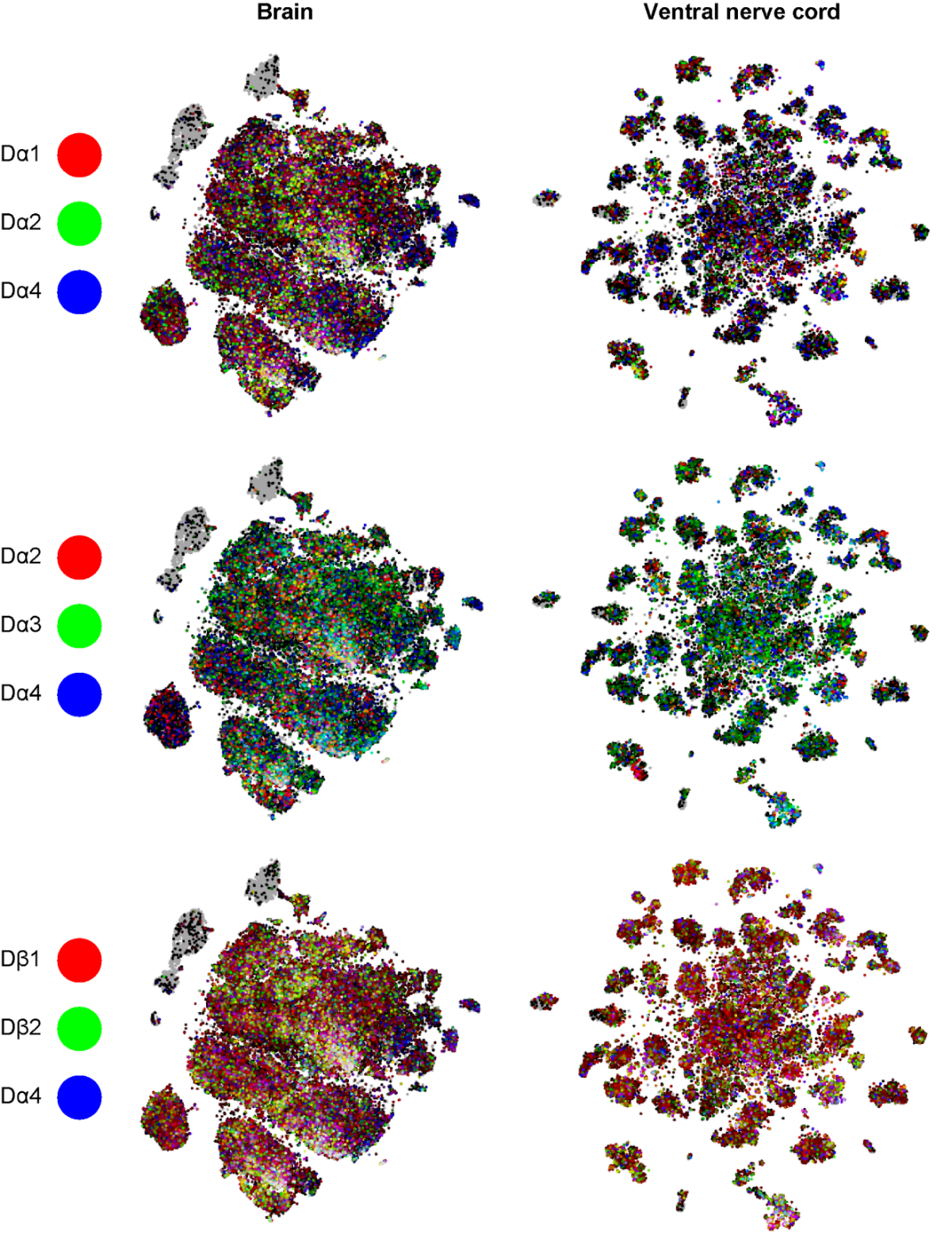


Fig D. t-SNE representations of *Dα1*, *Dα2*, *Dα3*, *Dα4*, *Dβ1*, and *Dβ2* gene expressions in the adult brain and ventral nerve cord of *D. melganogaster.* The figure was illustrated by SCope (https://scope.aertslab.org/#/86757313-d473-4f5f-b045-fc035d99451a/*/welcome) using single cell RNA-sequencing data [43]. These six nAChR subunit genes are co-expressed in single cells (See white dots.).

**
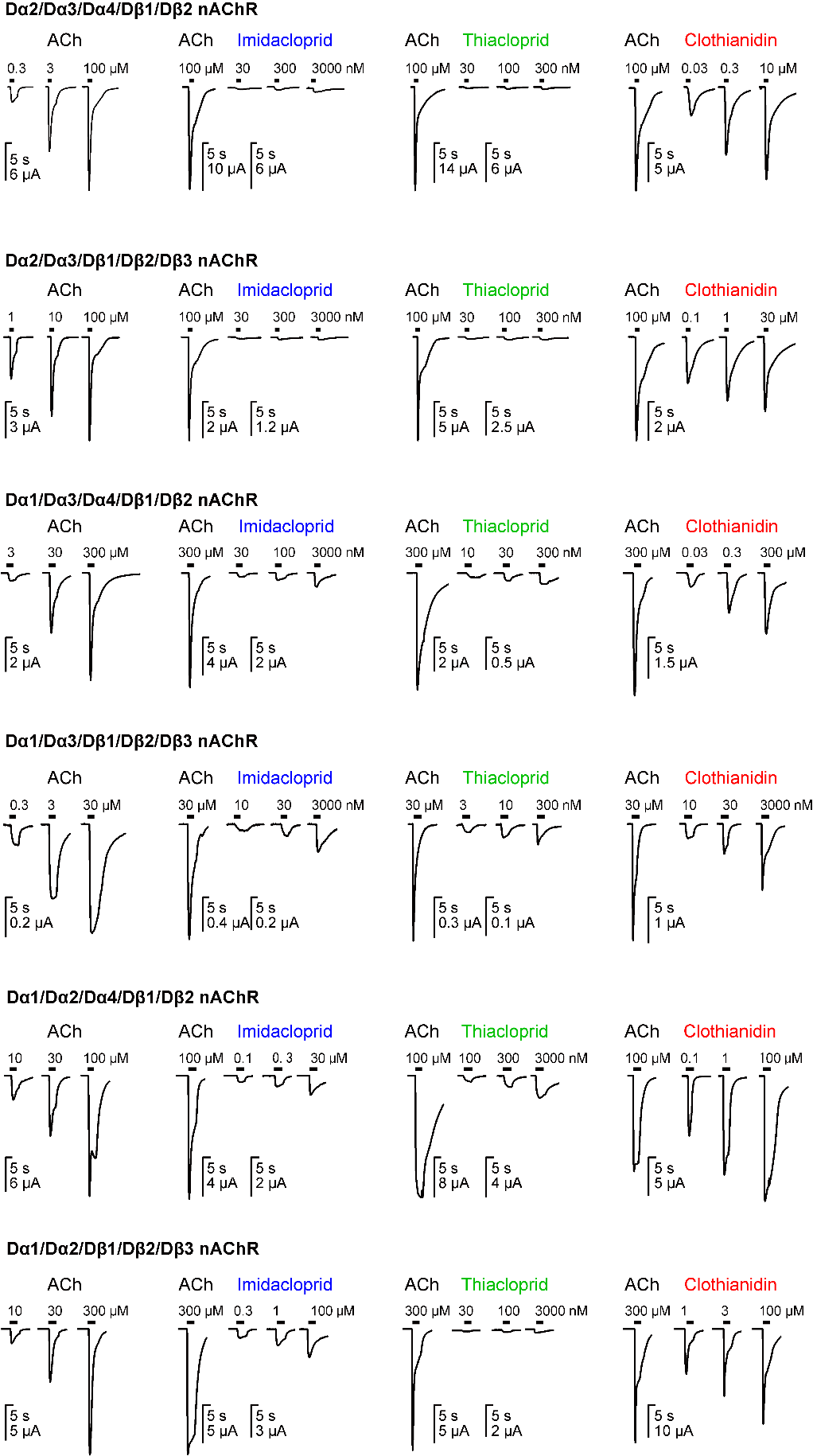
**

**Fig E.** Inward current responses to neonicotinoids (imidacloprid, thiacloprid, and clothianidin) of oocytes expressing *D. melanogaster* Dα2/Dα3/Dα4/Dβ1/Dβ2, Dα2/Dα3/Dβ1/Dβ2/Dβ3, Dα1/Dα3/Dα4/Dβ1/Dβ2, Dα1/Dα3/Dβ1/Dβ2/Dβ3, Dα1/Dα2/Dα4/Dβ1/Dβ2, and Dα1/Dα2/Dβ1/Dβ2/Dβ3 nAChRs. Horizontal lines indicate application of neonicotinoids.

Table A. One-way ANOVA of the pEC_50_ values of ACh for *D. melanogaster* nAChRs

| **nAChRs compared** | **Difference** | **95% CI of difference** | **Summary^†^** | **Adjusted *P* Value** |
| --- | --- | --- | --- | --- |
| Dα1/Dβ1 vs. Dα3/Dβ1 | -0.90 | -1.15 to -0.650 | **** | <0.0001 |
| Dα1/Dβ1 vs. Dα1/Dα3/Dβ1 | -0.61 | -0.860 to -0.360 | **** | <0.0001 |
| Dα1/Dβ1 vs. Dα1/Dα2/Dβ1 | 1.10 | 0.850 to 1.35 | **** | <0.0001 |
| Dα1/Dβ1 vs. Dα2/Dα3/Dβ1 | -0.21 | -0.460 to 0.0404 | ns | 0.1809 |
| Dα1/Dβ1 vs. Dα1/Dα2/Dα3/Dβ1 | -0.19 | -0.440 to 0.0604 | ns | 0.3051 |
| Dα1/Dβ1 vs. Dα1/Dβ1/Dβ2 | 0.49 | 0.240 to 0.740 | **** | <0.0001 |
| Dα1/Dβ1 vs. Dα3/Dβ1/Dβ2 | -0.95 | -1.20 to -0.700 | **** | <0.0001 |
| Dα1/Dβ1 vs. Dα1/Dα3/Dβ1/Dβ2 | -1.19 | -1.44 to -0.940 | **** | <0.0001 |
| Dα1/Dβ1 vs. Dα1/Dα2/Dβ1/Dβ2 | 0.32 | 0.0696 to 0.570 | ** | 0.0033 |
| Dα1/Dβ1 vs. Dα2/Dα3/Dβ1/Dβ2 | -0.75 | -1.00 to -0.500 | **** | <0.0001 |
| Dα1/Dβ1 vs. Dα1/Dα2/Dα3/Dβ1/Dβ2 | 0.75 | 0.500 to 1.00 | **** | <0.0001 |
| Dα3/Dβ1 vs. Dα1/Dα3/Dβ1 | 0.29 | 0.0396 to 0.540 | * | 0.0112 |
| Dα3/Dβ1 vs. Dα1/Dα2/Dβ1 | 2.00 | 1.75 to 2.25 | **** | <0.0001 |
| Dα3/Dβ1 vs. Dα2/Dα3/Dβ1 | 0.69 | 0.440 to 0.940 | **** | <0.0001 |
| Dα3/Dβ1 vs. Dα1/Dα2/Dα3/Dβ1 | 0.71 | 0.460 to 0.960 | **** | <0.0001 |
| Dα3/Dβ1 vs. Dα1/Dβ1/Dβ2 | 1.39 | 1.14 to 1.64 | **** | <0.0001 |
| Dα3/Dβ1 vs. Dα3/Dβ1/Dβ2 | -0.05 | -0.300 to 0.200 | ns | >0.9999 |
| Dα3/Dβ1 vs. Dα1/Dα3/Dβ1/Dβ2 | -0.29 | -0.540 to -0.0396 | * | 0.0112 |
| Dα3/Dβ1 vs. Dα1/Dα2/Dβ1/Dβ2 | 1.22 | 0.970 to 1.47 | **** | <0.0001 |
| Dα3/Dβ1 vs. Dα2/Dα3/Dβ1/Dβ2 | 0.15 | -0.100 to 0.400 | ns | 0.6537 |
| Dα3/Dβ1 vs. Dα1/Dα2/Dα3/Dβ1/Dβ2 | 1.65 | 1.40 to 1.90 | **** | <0.0001 |
| Dα1/Dα3/Dβ1 vs. Dα1/Dα2/Dβ1 | 1.71 | 1.46 to 1.96 | **** | <0.0001 |
| Dα1/Dα3/Dβ1 vs. Dα2/Dα3/Dβ1 | 0.40 | 0.150 to 0.650 | **** | <0.0001 |
| Dα1/Dα3/Dβ1 vs. Dα1/Dα2/Dα3/Dβ1 | 0.42 | 0.170 to 0.670 | **** | <0.0001 |
| Dα1/Dα3/Dβ1 vs. Dα1/Dβ1/Dβ2 | 1.10 | 0.850 to 1.35 | **** | <0.0001 |
| Dα1/Dα3/Dβ1 vs. Dα3/Dβ1/Dβ2 | -0.34 | -0.590 to -0.0896 | ** | 0.0014 |
| Dα1/Dα3/Dβ1 vs. Dα1/Dα3/Dβ1/Dβ2 | -0.58 | -0.830 to -0.330 | **** | <0.0001 |
| Dα1/Dα3/Dβ1 vs. Dα1/Dα2/Dβ1/Dβ2 | 0.93 | 0.680 to 1.18 | **** | <0.0001 |
| Dα1/Dα3/Dβ1 vs. Dα2/Dα3/Dβ1/Dβ2 | -0.14 | -0.390 to 0.110 | ns | 0.7412 |
| Dα1/Dα3/Dβ1 vs. Dα1/Dα2/Dα3/Dβ1/Dβ2 | 1.36 | 1.11 to 1.61 | **** | <0.0001 |
| Dα1/Dα2/Dβ1 vs. Dα2/Dα3/Db1 | -1.31 | -1.56 to -1.06 | **** | <0.0001 |
| Dα1/Dα2/Dβ1 vs. Dα1/Dα2/Dα3/Db1 | -1.29 | -1.54 to -1.04 | **** | <0.0001 |
| Dα1/Dα2/Dβ1 vs. Dα1/Dβ1/Dβ2 | -0.61 | -0.860 to -0.360 | **** | <0.0001 |
| Dα1/Dα2/Dβ1 vs. Dα3/ Dβ1/Dβ2 | -2.05 | -2.30 to -1.80 | **** | <0.0001 |
| Dα1/Dα2/Dβ1 vs. Dα1/Dα3/Dβ1/Dβ2 | -2.29 | -2.54 to -2.04 | **** | <0.0001 |
| Dα1/Dα2/Dβ1 vs. Dα1/Dα2/Dβ1/Dβ2 | -0.78 | -1.03 to -0.530 | **** | <0.0001 |
| Dα1/Dα2/Dβ1 vs. Dα2/Dα3/Dβ1/Dβ2 | -1.85 | -2.10 to -1.60 | **** | <0.0001 |
| Dα1/Dα2/Dβ1 vs. Dα1/Dα2/Dα3/Dβ1/Dβ2 | -0.35 | -0.600 to -0.0996 | *** | 0.0009 |
| Dα2/Dα3/Dβ1 vs. Dα1/Dα2/Dα3/Dβ1 | 0.02 | -0.230 to 0.270 | ns | >0.9999 |
| Dα2/Dα3/Dβ1 vs. Dα1/Dβ1/Dβ2 | 0.70 | 0.450 to 0.950 | **** | <0.0001 |
| Dα2/Dα3/Dβ1 vs. Dα3/ Dβ1/Dβ2 | -0.74 | -0.990 to -0.490 | **** | <0.0001 |
| Dα2/Dα3/Dβ1 vs. Dα1/Dα3/Dβ1/Dβ2 | -0.98 | -1.23 to -0.730 | **** | <0.0001 |
| Dα2/Dα3/Dβ1 vs. Dα1/Dα2/Dβ1/Dβ2 | 0.53 | 0.280 to 0.780 | **** | <0.0001 |
| Dα2/Dα3/Dβ1 vs. Dα2/Dα3/Dβ1/Dβ2 | -0.54 | -0.790 to -0.290 | **** | <0.0001 |
| Dα2/Dα3/Dβ1 vs. Dα1/Dα2/Da3/Dβ1/Dβ2 | 0.96 | 0.710 to 1.21 | **** | <0.0001 |
| Dα1/Dα2/Dα3/Dβ1 vs. Dα1/Dβ1/Dβ2 | 0.68 | 0.430 to 0.930 | **** | <0.0001 |
| Dα1/Dα2/Dα3/Dβ1 vs. Dα3/Dβ1/Dβ2 | -0.76 | -1.01 to -0.510 | **** | <0.0001 |
| Dα1/Dα2/Dα3/Dβ1 vs. Dα1/Dα3/Dβ1/Dβ2 | -1.00 | -1.25 to -0.750 | **** | <0.0001 |
| Dα1/Dα2/Dα3/Dβ1 vs. Dα1/Dα2/Dβ1/Dβ2 | 0.51 | 0.260 to 0.760 | **** | <0.0001 |
| Dα1/Dα2/Dα3/Dβ1 vs. Dα2/Dα3/Dβ1/Dβ2 | -0.56 | -0.810 to -0.310 | **** | <0.0001 |
| Dα1/Dα2/Dα3/Dβ1 vs. Dα1/Dα2/Dα3/Dβ1/Dβ2 | 0.94 | 0.690 to 1.19 | **** | <0.0001 |
| Dα1/Dβ1/Dβ2 vs. Dα3/Dβ1/Dβ2 | -1.44 | -1.69 to -1.19 | **** | <0.0001 |
| Dα1/Dβ1/Dβ2 vs. Dα1/Dα3/ Dβ1/Dβ2 | -1.68 | -1.93 to -1.43 | **** | <0.0001 |
| Dα1/Dβ1/Dβ2 vs. Dα1/Dα2/ Dβ1/Dβ2 | -0.17 | -0.420 to 0.0804 | ns | 0.4698 |
| Dα1/Dβ1/Dβ2 vs. Dα2/Dα3/ Dβ1/Dβ2 | -1.24 | -1.49 to -0.990 | **** | <0.0001 |
| Dα1/Dβ1/Dβ2 vs. Dα1/Dα2/Da3/ Dβ1/Dβ2 | 0.26 | 0.00963 to 0.510 | * | 0.0354 |
| Dα3/Dβ1/Dβ2 vs. Dα1/Dα3/ Dβ1/Dβ2 | -0.24 | -0.490 to 0.0104 | ns | 0.0714 |
| Dα3/Dβ1/Dβ2 vs. Dα1/Dα2/ Dβ1/Dβ2 | 1.27 | 1.02 to 1.52 | **** | <0.0001 |
| Dα3/Dβ1/Dβ2 vs. Dα2/Dα3/ Dβ1/Dβ2 | 0.20 | -0.0504 to 0.450 | ns | 0.2375 |
| Dα3/Dβ1/Dβ2 vs. Dα1/Dα2/Dα3/Dβ1/Dβ2 | 1.70 | 1.45 to 1.95 | **** | <0.0001 |
| Dα1/Dα3/Dβ1/Dβ2 vs. Dα1/Dα2/Dβ1/Dβ2 | 1.51 | 1.26 to 1.76 | **** | <0.0001 |
| Dα1/Dα3/Dβ1/Dβ2 vs. Dα2/Dα3/Dβ1/Dβ2 | 0.44 | 0.190 to 0.690 | **** | <0.0001 |
| Dα1/Dα3/Dβ1/Dβ2 vs. Dα1/Dα2/Da3/Dβ1/Dβ2 | 1.94 | 1.69 to 2.19 | **** | <0.0001 |
| Dα1/Dα2/Dβ1/Dβ2 vs. Dα2/Dα3/Dβ1/Dβ2 | -1.07 | -1.32 to -0.820 | **** | <0.0001 |
| Dα1/Dα2/Dβ1/Dβ2 vs. Dα1/Dα2/Dα3/Dβ1/Dβ2 | 0.43 | 0.180 to 0.680 | **** | <0.0001 |
| Dα2/Dα3/Dβ1/Dβ2 vs. Dα1/Dα2/Dα3/Dβ1/Dβ2 | 1.50 | 1.25 to 1.75 | **** | <0.0001 |

**^†^**One-way ANOVA, Tukey test, *, *P* < 0.05; **, *P* < 0.01; *****, *P* < 0.001; ****, *P* < 0.0001; ns, not significant.

Table B. One-way ANOVA of the pEC_50_ values of imidacloprid for *D. melanogaster* nAChRs

| **nAChRs compared** | **Difference** | **95% CI of difference** | **Summary^†^** | **Adjusted *P* Value** |
| --- | --- | --- | --- | --- |
| Dα1/Dβ1 vs. Dα3/Dβ1 | -1.16 | -2.10 to -0.221 | ** | 0.0051 |
| Dα1/Dβ1 vs. Dα1/Dα3/Dβ1 | -0.42 | -1.36 to 0.519 | ns | 0.9225 |
| Dα1/Dβ1 vs. Dα1/Dα2/Dβ1 | 0.45 | -0.489 to 1.39 | ns | 0.8827 |
| Dα1/Dβ1 vs. Dα2/Dα3/Dβ1 | 0.28 | -0.659 to 1.22 | ns | 0.9963 |
| Dα1/Dβ1 vs. Dα1/Dα2/Dα3/Dβ1 | 0.08 | -0.859 to 1.02 | ns | >0.9999 |
| Dα1/Dβ1 vs. Dα1/Dβ1/Dβ2 | 0.17 | -0.769 to 1.11 | ns | >0.9999 |
| Dα1/Dβ1 vs. Dα3/Dβ1/Dβ2 | -1.12 | -2.06 to -0.181 | ** | 0.0079 |
| Dα1/Dβ1 vs. Dα1/Dα3/Dβ1/Dβ2 | -0.36 | -1.30 to 0.579 | ns | 0.9727 |
| Dα1/Dβ1 vs. Dα1/Dα2/Dβ1/Dβ2 | 0.71 | -0.229 to 1.65 | ns | 0.3097 |
| Dα1/Dβ1 vs. Dα2/Dα3/Dβ1/Dβ2 | 0.16 | -0.779 to 1.10 | ns | >0.9999 |
| Dα1/Dβ1 vs. Dα1/Dα2/Dα3/Dβ1/Dβ2 | 0.69 | -0.249 to 1.63 | ns | 0.3505 |
| Dα3/Dβ1 vs. Dα1/Dα3/Dβ1 | 0.74 | -0.199 to 1.68 | ns | 0.2542 |
| Dα3/Dβ1 vs. Dα1/Dα2/Dβ1 | 1.61 | 0.671 to 2.55 | **** | <0.0001 |
| Dα3/Dβ1 vs. Dα2/Dα3/Dβ1 | 1.44 | 0.501 to 2.38 | *** | 0.0002 |
| Dα3/Dβ1 vs. Dα1/Dα2/Dα3/Dβ1 | 1.24 | 0.301 to 2.18 | ** | 0.0021 |
| Dα3/Dβ1 vs. Dα1/Dβ1/Dβ2 | 1.33 | 0.391 to 2.27 | *** | 0.0007 |
| Dα3/Dβ1 vs. Dα3/Dβ1/Dβ2 | 0.04 | -0.899 to 0.979 | ns | >0.9999 |
| Dα3/Dβ1 vs. Dα1/Dα3/Dβ1/Dβ2 | 0.80 | -0.139 to 1.74 | ns | 0.1643 |
| Dα3/Dβ1 vs. Dα1/Dα2/Dβ1/Dβ2 | 1.87 | 0.931 to 2.81 | **** | <0.0001 |
| Dα3/Dβ1 vs. Dα2/Dα3/Dβ1/Dβ2 | 1.32 | 0.381 to 2.26 | *** | 0.0008 |
| Dα3/Dβ1 vs. Dα1/Dα2/Dα3/Dβ1/Dβ2 | 1.85 | 0.911 to 2.79 | **** | <0.0001 |
| Dα1/Dα3/Dβ1 vs. Dα1/Dα2/Dβ1 | 0.87 | -0.0687 to 1.81 | ns | 0.0927 |
| Dα1/Dα3/Dβ1 vs. Dα2/Dα3/Dβ1 | 0.70 | -0.239 to 1.64 | ns | 0.3298 |
| Dα1/Dα3/Dβ1 vs. Dα1/Dα2/Dα3/Dβ1 | 0.50 | -0.439 to 1.44 | ns | 0.7942 |
| Dα1/Dα3/Dβ1 vs. Dα1/Dβ1/Dβ2 | 0.59 | -0.349 to 1.53 | ns | 0.5859 |
| Dα1/Dα3/Dβ1 vs. Dα3/Dβ1/Dβ2 | -0.70 | -1.64 to 0.239 | ns | 0.3298 |
| Dα1/Dα3/Dβ1 vs. Dα1/Dα3/Dβ1/Dβ2 | 0.06 | -0.879 to 0.999 | ns | >0.9999 |
| Dα1/Dα3/Dβ1 vs. Dα1/Dα2/Dβ1/Dβ2 | 1.13 | 0.191 to 2.07 | ** | 0.0071 |
| Dα1/Dα3/Dβ1 vs. Dα2/Dα3/Dβ1/Dβ2 | 0.58 | -0.359 to 1.52 | ns | 0.6106 |
| Dα1/Dα3/Dβ1 vs. Dα1/Dα2/Dα3/Dβ1/Dβ2 | 1.11 | 0.171 to 2.05 | ** | 0.0088 |
| Dα1/Dα2/Dβ1 vs. Dα2/Dα3/Db1 | -0.17 | -1.11 to 0.769 | ns | >0.9999 |
| Dα1/Dα2/Dβ1 vs. Dα1/Dα2/Dα3/Db1 | -0.37 | -1.31 to 0.569 | ns | 0.9668 |
| Dα1/Dα2/Dβ1 vs. Dα1/Dβ1/Dβ2 | -0.28 | -1.22 to 0.659 | ns | 0.9963 |
| Dα1/Dα2/Dβ1 vs. Dα3/ Dβ1/Dβ2 | -1.57 | -2.51 to -0.631 | **** | <0.0001 |
| Dα1/Dα2/Dβ1 vs. Dα1/Dα3/Dβ1/Dβ2 | -0.81 | -1.75 to 0.129 | ns | 0.152 |
| Dα1/Dα2/Dβ1 vs. Dα1/Dα2/Dβ1/Dβ2 | 0.26 | -0.679 to 1.20 | ns | 0.9981 |
| Dα1/Dα2/Dβ1 vs. Dα2/Dα3/Dβ1/Dβ2 | -0.29 | -1.23 to 0.649 | ns | 0.995 |
| Dα1/Dα2/Dβ1 vs. Dα1/Dα2/Dα3/Dβ1/Dβ2 | 0.24 | -0.699 to 1.18 | ns | 0.9991 |
| Dα2/Dα3/Dβ1 vs. Dα1/Dα2/Dα3/Dβ1 | -0.20 | -1.14 to 0.739 | ns | 0.9998 |
| Dα2/Dα3/Dβ1 vs. Dα1/Dβ1/Dβ2 | -0.11 | -1.05 to 0.829 | ns | >0.9999 |
| Dα2/Dα3/Dβ1 vs. Dα3/ Dβ1/Dβ2 | -1.40 | -2.34 to -0.461 | *** | 0.0003 |
| Dα2/Dα3/Dβ1 vs. Dα1/Dα3/Dβ1/Dβ2 | -0.64 | -1.58 to 0.299 | ns | 0.4635 |
| Dα2/Dα3/Dβ1 vs. Dα1/Dα2/Dβ1/Dβ2 | 0.43 | -0.509 to 1.37 | ns | 0.9103 |
| Dα2/Dα3/Dβ1 vs. Dα2/Dα3/Dβ1/Dβ2 | -0.12 | -1.06 to 0.819 | ns | >0.9999 |
| Dα2/Dα3/Dβ1 vs. Dα1/Dα2/Da3/Dβ1/Dβ2 | 0.41 | -0.529 to 1.35 | ns | 0.9334 |
| Dα1/Dα2/Dα3/Dβ1 vs. Dα1/Dβ1/Dβ2 | 0.09 | -0.849 to 1.03 | ns | >0.9999 |
| Dα1/Dα2/Dα3/Dβ1 vs. Dα3/Dβ1/Dβ2 | -1.20 | -2.14 to -0.261 | ** | 0.0032 |
| Dα1/Dα2/Dα3/Dβ1 vs. Dα1/Dα3/Dβ1/Dβ2 | -0.44 | -1.38 to 0.499 | ns | 0.8971 |
| Dα1/Dα2/Dα3/Dβ1 vs. Dα1/Dα2/Dβ1/Dβ2 | 0.63 | -0.309 to 1.57 | ns | 0.4875 |
| Dα1/Dα2/Dα3/Dβ1 vs. Dα2/Dα3/Dβ1/Dβ2 | 0.08 | -0.859 to 1.02 | ns | >0.9999 |
| Dα1/Dα2/Dα3/Dβ1 vs. Dα1/Dα2/Dα3/Dβ1/Dβ2 | 0.61 | -0.329 to 1.55 | ns | 0.5364 |
| Dα1/Dβ1/Dβ2 vs. Dα3/Dβ1/Dβ2 | -1.29 | -2.23 to -0.351 | ** | 0.0011 |
| Dα1/Dβ1/Dβ2 vs. Dα1/Dα3/ Dβ1/Dβ2 | -0.53 | -1.47 to 0.409 | ns | 0.7298 |
| Dα1/Dβ1/Dβ2 vs. Dα1/Dα2/ Dβ1/Dβ2 | 0.54 | -0.399 to 1.48 | ns | 0.7069 |
| Dα1/Dβ1/Dβ2 vs. Dα2/Dα3/ Dβ1/Dβ2 | -0.01 | -0.949 to 0.929 | ns | >0.9999 |
| Dα1/Dβ1/Dβ2 vs. Dα1/Dα2/Da3/ Dβ1/Dβ2 | 0.52 | -0.419 to 1.46 | ns | 0.752 |
| Dα3/Dβ1/Dβ2 vs. Dα1/Dα3/ Dβ1/Dβ2 | 0.76 | -0.179 to 1.70 | ns | 0.2211 |
| Dα3/Dβ1/Dβ2 vs. Dα1/Dα2/ Dβ1/Dβ2 | 1.83 | 0.891 to 2.77 | **** | <0.0001 |
| Dα3/Dβ1/Dβ2 vs. Dα2/Dα3/ Dβ1/Dβ2 | 1.28 | 0.341 to 2.22 | ** | 0.0013 |
| Dα3/Dβ1/Dβ2 vs. Dα1/Dα2/Dα3/Dβ1/Dβ2 | 1.81 | 0.871 to 2.75 | **** | <0.0001 |
| Dα1/Dα3/Dβ1/Dβ2 vs. Dα1/Dα2/Dβ1/Dβ2 | 1.07 | 0.131 to 2.01 | * | 0.0135 |
| Dα1/Dα3/Dβ1/Dβ2 vs. Dα2/Dα3/Dβ1/Dβ2 | 0.52 | -0.419 to 1.46 | ns | 0.752 |
| Dα1/Dα3/Dβ1/Dβ2 vs. Dα1/Dα2/Da3/Dβ1/Dβ2 | 1.05 | 0.111 to 1.99 | * | 0.0166 |
| Dα1/Dα2/Dβ1/Dβ2 vs. Dα2/Dα3/Dβ1/Dβ2 | -0.55 | -1.49 to 0.389 | ns | 0.6834 |
| Dα1/Dα2/Dβ1/Dβ2 vs. Dα1/Dα2/Dα3/Dβ1/Dβ2 | -0.02 | -0.959 to 0.919 | ns | >0.9999 |
| Dα2/Dα3/Dβ1/Dβ2 vs. Dα1/Dα2/Dα3/Dβ1/Dβ2 | 0.53 | -0.409 to 1.47 | ns | 0.7298 |

**^†^**One-way ANOVA, Tukey test, *, *P* < 0.05; **, *P* < 0.01; *****, *P* < 0.001; ****, *P* < 0.0001; ns, not significant.

Table C. One-way ANOVA of the I_max_ values of imidacloprid for *D. melanogaster* nAChRs

| **nAChRs compared** | **Difference** | **95% CI of difference** | **Summary^†^** | **Adjusted *P* Value** |
| --- | --- | --- | --- | --- |
| Dα1/Dβ1 vs. Dα3/Dβ1 | 0.090 | 0.0300 to 0.150 | *** | 0.0003 |
| Dα1/Dβ1 vs. Dα1/Dα3/Dβ1 | -0.048 | -0.108 to 0.0120 | ns | 0.2356 |
| Dα1/Dβ1 vs. Dα1/Dα2/Dβ1 | 0.091 | 0.0310 to 0.151 | *** | 0.0002 |
| Dα1/Dβ1 vs. Dα2/Dα3/Dβ1 | 0.021 | -0.0390 to 0.0810 | ns | 0.9863 |
| Dα1/Dβ1 vs. Dα1/Dα2/Dα3/Dβ1 | 0.028 | -0.0320 to 0.0880 | ns | 0.8997 |
| Dα1/Dβ1 vs. Dα1/Dβ1/Dβ2 | 0.046 | -0.0140 to 0.106 | ns | 0.2911 |
| Dα1/Dβ1 vs. Dα3/Dβ1/Dβ2 | 0.083 | 0.0230 to 0.143 | ** | 0.001 |
| Dα1/Dβ1 vs. Dα1/Dα3/Dβ1/Dβ2 | -0.208 | -0.268 to -0.148 | **** | <0.0001 |
| Dα1/Dβ1 vs. Dα1/Dα2/Dβ1/Dβ2 | -0.043 | -0.103 to 0.0170 | ns | 0.3879 |
| Dα1/Dβ1 vs. Dα2/Dα3/Dβ1/Dβ2 | -0.003 | -0.0630 to 0.0570 | ns | >0.9999 |
| Dα1/Dβ1 vs. Dα1/Dα2/Dα3/Dβ1/Dβ2 | -0.020 | -0.0800 to 0.0400 | ns | 0.9907 |
| Dα3/Dβ1 vs. Dα1/Dα3/Dβ1 | -0.138 | -0.198 to -0.0780 | **** | <0.0001 |
| Dα3/Dβ1 vs. Dα1/Dα2/Dβ1 | 0.001 | -0.0590 to 0.0610 | ns | >0.9999 |
| Dα3/Dβ1 vs. Dα2/Dα3/Dβ1 | -0.069 | -0.129 to -0.00900 | * | 0.0122 |
| Dα3/Dβ1 vs. Dα1/Dα2/Dα3/Dβ1 | -0.062 | -0.122 to -0.00200 | * | 0.0371 |
| Dα3/Dβ1 vs. Dα1/Dβ1/Dβ2 | -0.044 | -0.104 to 0.0160 | ns | 0.354 |
| Dα3/Dβ1 vs. Dα3/Dβ1/Dβ2 | -0.007 | -0.0670 to 0.0530 | ns | >0.9999 |
| Dα3/Dβ1 vs. Dα1/Dα3/Dβ1/Dβ2 | -0.298 | -0.358 to -0.238 | **** | <0.0001 |
| Dα3/Dβ1 vs. Dα1/Dα2/Dβ1/Dβ2 | -0.133 | -0.193 to -0.0730 | **** | <0.0001 |
| Dα3/Dβ1 vs. Dα2/Dα3/Dβ1/Dβ2 | -0.093 | -0.153 to -0.0330 | *** | 0.0002 |
| Dα3/Dβ1 vs. Dα1/Dα2/Dα3/Dβ1/Dβ2 | -0.110 | -0.170 to -0.0500 | **** | <0.0001 |
| Dα1/Dα3/Dβ1 vs. Dα1/Dα2/Dβ1 | 0.139 | 0.0790 to 0.199 | **** | <0.0001 |
| Dα1/Dα3/Dβ1 vs. Dα2/Dα3/Dβ1 | 0.069 | 0.00900 to 0.129 | * | 0.0122 |
| Dα1/Dα3/Dβ1 vs. Dα1/Dα2/Dα3/Dβ1 | 0.076 | 0.0160 to 0.136 | ** | 0.0037 |
| Dα1/Dα3/Dβ1 vs. Dα1/Dβ1/Dβ2 | 0.094 | 0.0340 to 0.154 | *** | 0.0001 |
| Dα1/Dα3/Dβ1 vs. Dα3/Dβ1/Dβ2 | 0.131 | 0.0710 to 0.191 | **** | <0.0001 |
| Dα1/Dα3/Dβ1 vs. Dα1/Dα3/Dβ1/Dβ2 | -0.160 | -0.220 to -0.100 | **** | <0.0001 |
| Dα1/Dα3/Dβ1 vs. Dα1/Dα2/Dβ1/Dβ2 | 0.005 | -0.0550 to 0.0650 | ns | >0.9999 |
| Dα1/Dα3/Dβ1 vs. Dα2/Dα3/Dβ1/Dβ2 | 0.045 | -0.0150 to 0.105 | ns | 0.3216 |
| Dα1/Dα3/Dβ1 vs. Dα1/Dα2/Dα3/Dβ1/Dβ2 | 0.028 | -0.0320 to 0.0880 | ns | 0.8997 |
| Dα1/Dα2/Dβ1 vs. Dα2/Dα3/Db1 | -0.070 | -0.130 to -0.0100 | * | 0.0103 |
| Dα1/Dα2/Dβ1 vs. Dα1/Dα2/Dα3/Db1 | -0.063 | -0.123 to -0.00300 | * | 0.0319 |
| Dα1/Dα2/Dβ1 vs. Dα1/Dβ1/Dβ2 | -0.045 | -0.105 to 0.0150 | ns | 0.3216 |
| Dα1/Dα2/Dβ1 vs. Dα3/ Dβ1/Dβ2 | -0.008 | -0.0680 to 0.0520 | ns | >0.9999 |
| Dα1/Dα2/Dβ1 vs. Dα1/Dα3/Dβ1/Dβ2 | -0.299 | -0.359 to -0.239 | **** | <0.0001 |
| Dα1/Dα2/Dβ1 vs. Dα1/Dα2/Dβ1/Dβ2 | -0.134 | -0.194 to -0.0740 | **** | <0.0001 |
| Dα1/Dα2/Dβ1 vs. Dα2/Dα3/Dβ1/Dβ2 | -0.094 | -0.154 to -0.0340 | *** | 0.0001 |
| Dα1/Dα2/Dβ1 vs. Dα1/Dα2/Dα3/Dβ1/Dβ2 | -0.111 | -0.171 to -0.0510 | **** | <0.0001 |
| Dα2/Dα3/Dβ1 vs. Dα1/Dα2/Dα3/Dβ1 | 0.007 | -0.0530 to 0.0670 | ns | >0.9999 |
| Dα2/Dα3/Dβ1 vs. Dα1/Dβ1/Dβ2 | 0.025 | -0.0350 to 0.0850 | ns | 0.9512 |
| Dα2/Dα3/Dβ1 vs. Dα3/ Dβ1/Dβ2 | 0.062 | 0.00200 to 0.122 | * | 0.0371 |
| Dα2/Dα3/Dβ1 vs. Dα1/Dα3/Dβ1/Dβ2 | -0.229 | -0.289 to -0.169 | **** | <0.0001 |
| Dα2/Dα3/Dβ1 vs. Dα1/Dα2/Dβ1/Dβ2 | -0.064 | -0.124 to -0.00400 | * | 0.0273 |
| Dα2/Dα3/Dβ1 vs. Dα2/Dα3/Dβ1/Dβ2 | -0.024 | -0.0840 to 0.0360 | ns | 0.9632 |
| Dα2/Dα3/Dβ1 vs. Dα1/Dα2/Da3/Dβ1/Dβ2 | -0.041 | -0.101 to 0.0190 | ns | 0.46 |
| Dα1/Dα2/Dα3/Dβ1 vs. Dα1/Dβ1/Dβ2 | 0.018 | -0.0420 to 0.0780 | ns | 0.9961 |
| Dα1/Dα2/Dα3/Dβ1 vs. Dα3/Dβ1/Dβ2 | 0.055 | -0.00500 to 0.115 | ns | 0.1006 |
| Dα1/Dα2/Dα3/Dβ1 vs. Dα1/Dα3/Dβ1/Dβ2 | -0.236 | -0.296 to -0.176 | **** | <0.0001 |
| Dα1/Dα2/Dα3/Dβ1 vs. Dα1/Dα2/Dβ1/Dβ2 | -0.071 | -0.131 to -0.0110 | ** | 0.0087 |
| Dα1/Dα2/Dα3/Dβ1 vs. Dα2/Dα3/Dβ1/Dβ2 | -0.031 | -0.0910 to 0.0290 | ns | 0.8235 |
| Dα1/Dα2/Dα3/Dβ1 vs. Dα1/Dα2/Dα3/Dβ1/Dβ2 | -0.048 | -0.108 to 0.0120 | ns | 0.2356 |
| Dα1/Dβ1/Dβ2 vs. Dα3/Dβ1/Dβ2 | 0.037 | -0.0230 to 0.0970 | ns | 0.6134 |
| Dα1/Dβ1/Dβ2 vs. Dα1/Dα3/ Dβ1/Dβ2 | -0.254 | -0.314 to -0.194 | **** | <0.0001 |
| Dα1/Dβ1/Dβ2 vs. Dα1/Dα2/ Dβ1/Dβ2 | -0.089 | -0.149 to -0.0290 | *** | 0.0003 |
| Dα1/Dβ1/Dβ2 vs. Dα2/Dα3/ Dβ1/Dβ2 | -0.049 | -0.109 to 0.0110 | ns | 0.2108 |
| Dα1/Dβ1/Dβ2 vs. Dα1/Dα2/Da3/ Dβ1/Dβ2 | -0.066 | -0.126 to -0.00600 | * | 0.0199 |
| Dα3/Dβ1/Dβ2 vs. Dα1/Dα3/ Dβ1/Dβ2 | -0.291 | -0.351 to -0.231 | **** | <0.0001 |
| Dα3/Dβ1/Dβ2 vs. Dα1/Dα2/ Dβ1/Dβ2 | -0.126 | -0.186 to -0.0660 | **** | <0.0001 |
| Dα3/Dβ1/Dβ2 vs. Dα2/Dα3/ Dβ1/Dβ2 | -0.086 | -0.146 to -0.0260 | *** | 0.0006 |
| Dα3/Dβ1/Dβ2 vs. Dα1/Dα2/Dα3/Dβ1/Dβ2 | -0.103 | -0.163 to -0.0430 | **** | <0.0001 |
| Dα1/Dα3/Dβ1/Dβ2 vs. Dα1/Dα2/Dβ1/Dβ2 | 0.165 | 0.105 to 0.225 | **** | <0.0001 |
| Dα1/Dα3/Dβ1/Dβ2 vs. Dα2/Dα3/Dβ1/Dβ2 | 0.205 | 0.145 to 0.265 | **** | <0.0001 |
| Dα1/Dα3/Dβ1/Dβ2 vs. Dα1/Dα2/Da3/Dβ1/Dβ2 | 0.188 | 0.128 to 0.248 | **** | <0.0001 |
| Dα1/Dα2/Dβ1/Dβ2 vs. Dα2/Dα3/Dβ1/Dβ2 | 0.040 | -0.0200 to 0.100 | ns | 0.4977 |
| Dα1/Dα2/Dβ1/Dβ2 vs. Dα1/Dα2/Dα3/Dβ1/Dβ2 | 0.023 | -0.0370 to 0.0830 | ns | 0.9728 |
| Dα2/Dα3/Dβ1/Dβ2 vs. Dα1/Dα2/Dα3/Dβ1/Dβ2 | -0.017 | -0.0770 to 0.0430 | ns | 0.9976 |

**^†^**One-way ANOVA, Tukey test, *, *P* < 0.05; **, *P* < 0.01; *****, *P* < 0.001; ****, *P* < 0.0001; ns, not significant.

Table D. One-way ANOVA of the pEC_50_ values of thiacloprid for *D. melanogaster* nAChRs

| **nAChRs compared** | **Difference** | **95% CI of difference** | **Summary^†^** | **Adjusted *P* Value** |
| --- | --- | --- | --- | --- |
| Dα1/Dβ1 vs. Dα3/Dβ1 | -0.01 | -1.01 to 0.995 | ns | >0.9999 |
| Dα1/Dβ1 vs. Dα1/Dα3/Dβ1 | -0.28 | -1.28 to 0.725 | ns | 0.998 |
| Dα1/Dβ1 vs. Dα1/Dα2/Dβ1 | 0.87 | -0.135 to 1.87 | ns | 0.1485 |
| Dα1/Dβ1 vs. Dα2/Dα3/Dβ1 | 0.87 | -0.135 to 1.87 | ns | 0.1485 |
| Dα1/Dβ1 vs. Dα1/Dα2/Dα3/Dβ1 | 0.06 | -0.945 to 1.06 | ns | >0.9999 |
| Dα1/Dβ1 vs. Dα1/Dβ1/Dβ2 | -0.07 | -1.07 to 0.935 | ns | >0.9999 |
| Dα1/Dβ1 vs. Dα3/Dβ1/Dβ2 | -0.53 | -1.53 to 0.475 | ns | 0.8037 |
| Dα1/Dβ1 vs. Dα1/Dα3/Dβ1/Dβ2 | -0.21 | -1.21 to 0.795 | ns | 0.9999 |
| Dα1/Dβ1 vs. Dα1/Dα2/Dβ1/Dβ2 | 0.69 | -0.315 to 1.69 | ns | 0.4521 |
| Dα1/Dβ1 vs. Dα2/Dα3/Dβ1/Dβ2 | -0.29 | -1.29 to 0.715 | ns | 0.9972 |
| Dα1/Dβ1 vs. Dα1/Dα2/Dα3/Dβ1/Dβ2 | 0.61 | -0.395 to 1.61 | ns | 0.6351 |
| Dα3/Dβ1 vs. Dα1/Dα3/Dβ1 | -0.27 | -1.27 to 0.735 | ns | 0.9985 |
| Dα3/Dβ1 vs. Dα1/Dα2/Dβ1 | 0.88 | -0.125 to 1.88 | ns | 0.1378 |
| Dα3/Dβ1 vs. Dα2/Dα3/Dβ1 | 0.88 | -0.125 to 1.88 | ns | 0.1378 |
| Dα3/Dβ1 vs. Dα1/Dα2/Dα3/Dβ1 | 0.07 | -0.935 to 1.07 | ns | >0.9999 |
| Dα3/Dβ1 vs. Dα1/Dβ1/Dβ2 | -0.06 | -1.06 to 0.945 | ns | >0.9999 |
| Dα3/Dβ1 vs. Dα3/Dβ1/Dβ2 | -0.52 | -1.52 to 0.485 | ns | 0.8217 |
| Dα3/Dβ1 vs. Dα1/Dα3/Dβ1/Dβ2 | -0.20 | -1.20 to 0.805 | ns | >0.9999 |
| Dα3/Dβ1 vs. Dα1/Dα2/Dβ1/Dβ2 | 0.70 | -0.305 to 1.70 | ns | 0.4302 |
| Dα3/Dβ1 vs. Dα2/Dα3/Dβ1/Dβ2 | -0.28 | -1.28 to 0.725 | ns | 0.998 |
| Dα3/Dβ1 vs. Dα1/Dα2/Dα3/Dβ1/Dβ2 | 0.62 | -0.385 to 1.62 | ns | 0.6122 |
| Dα1/Dα3/Dβ1 vs. Dα1/Dα2/Dβ1 | 1.15 | 0.145 to 2.15 | * | 0.0129 |
| Dα1/Dα3/Dβ1 vs. Dα2/Dα3/Dβ1 | 1.15 | 0.145 to 2.15 | * | 0.0129 |
| Dα1/Dα3/Dβ1 vs. Dα1/Dα2/Dα3/Dβ1 | 0.34 | -0.665 to 1.34 | ns | 0.9895 |
| Dα1/Dα3/Dβ1 vs. Dα1/Dβ1/Dβ2 | 0.21 | -0.795 to 1.21 | ns | 0.9999 |
| Dα1/Dα3/Dβ1 vs. Dα3/Dβ1/Dβ2 | -0.25 | -1.25 to 0.755 | ns | 0.9993 |
| Dα1/Dα3/Dβ1 vs. Dα1/Dα3/Dβ1/Dβ2 | 0.07 | -0.935 to 1.07 | ns | >0.9999 |
| Dα1/Dα3/Dβ1 vs. Dα1/Dα2/Dβ1/Dβ2 | 0.97 | -0.0345 to 1.97 | ns | 0.0673 |
| Dα1/Dα3/Dβ1 vs. Dα2/Dα3/Dβ1/Dβ2 | -0.01 | -1.01 to 0.995 | ns | >0.9999 |
| Dα1/Dα3/Dβ1 vs. Dα1/Dα2/Dα3/Dβ1/Dβ2 | 0.89 | -0.115 to 1.89 | ns | 0.1278 |
| Dα1/Dα2/Dβ1 vs. Dα2/Dα3/Db1 | 0.00 | -1.00 to 1.00 | ns | >0.9999 |
| Dα1/Dα2/Dβ1 vs. Dα1/Dα2/Dα3/Db1 | -0.81 | -1.81 to 0.195 | ns | 0.226 |
| Dα1/Dα2/Dβ1 vs. Dα1/Dβ1/Dβ2 | -0.94 | -1.94 to 0.0645 | ns | 0.0862 |
| Dα1/Dα2/Dβ1 vs. Dα3/ Dβ1/Dβ2 | -1.40 | -2.40 to -0.395 | *** | 0.0009 |
| Dα1/Dα2/Dβ1 vs. Dα1/Dα3/Dβ1/Dβ2 | -1.08 | -2.08 to -0.0755 | * | 0.0252 |
| Dα1/Dα2/Dβ1 vs. Dα1/Dα2/Dβ1/Dβ2 | -0.18 | -1.18 to 0.825 | ns | >0.9999 |
| Dα1/Dα2/Dβ1 vs. Dα2/Dα3/Dβ1/Dβ2 | -1.16 | -2.16 to -0.155 | * | 0.0116 |
| Dα1/Dα2/Dβ1 vs. Dα1/Dα2/Dα3/Dβ1/Dβ2 | -0.26 | -1.26 to 0.745 | ns | 0.9989 |
| Dα2/Dα3/Dβ1 vs. Dα1/Dα2/Dα3/Dβ1 | -0.81 | -1.81 to 0.195 | ns | 0.226 |
| Dα2/Dα3/Dβ1 vs. Dα1/Dβ1/Dβ2 | -0.94 | -1.94 to 0.0645 | ns | 0.0862 |
| Dα2/Dα3/Dβ1 vs. Dα3/ Dβ1/Dβ2 | -1.40 | -2.40 to -0.395 | *** | 0.0009 |
| Dα2/Dα3/Dβ1 vs. Dα1/Dα3/Dβ1/Dβ2 | -1.08 | -2.08 to -0.0755 | * | 0.0252 |
| Dα2/Dα3/Dβ1 vs. Dα1/Dα2/Dβ1/Dβ2 | -0.18 | -1.18 to 0.825 | ns | >0.9999 |
| Dα2/Dα3/Dβ1 vs. Dα2/Dα3/Dβ1/Dβ2 | -1.16 | -2.16 to -0.155 | * | 0.0116 |
| Dα2/Dα3/Dβ1 vs. Dα1/Dα2/Da3/Dβ1/Dβ2 | -0.26 | -1.26 to 0.745 | ns | 0.9989 |
| Dα1/Dα2/Dα3/Dβ1 vs. Dα1/Dβ1/Dβ2 | -0.13 | -1.13 to 0.875 | ns | >0.9999 |
| Dα1/Dα2/Dα3/Dβ1 vs. Dα3/Dβ1/Dβ2 | -0.59 | -1.59 to 0.415 | ns | 0.6802 |
| Dα1/Dα2/Dα3/Dβ1 vs. Dα1/Dα3/Dβ1/Dβ2 | -0.27 | -1.27 to 0.735 | ns | 0.9985 |
| Dα1/Dα2/Dα3/Dβ1 vs. Dα1/Dα2/Dβ1/Dβ2 | 0.63 | -0.375 to 1.63 | ns | 0.5891 |
| Dα1/Dα2/Dα3/Dβ1 vs. Dα2/Dα3/Dβ1/Dβ2 | -0.35 | -1.35 to 0.655 | ns | 0.9868 |
| Dα1/Dα2/Dα3/Dβ1 vs. Dα1/Dα2/Dα3/Dβ1/Dβ2 | 0.55 | -0.455 to 1.55 | ns | 0.7651 |
| Dα1/Dβ1/Dβ2 vs. Dα3/Dβ1/Dβ2 | -0.46 | -1.46 to 0.545 | ns | 0.9105 |
| Dα1/Dβ1/Dβ2 vs. Dα1/Dα3/ Dβ1/Dβ2 | -0.14 | -1.14 to 0.865 | ns | >0.9999 |
| Dα1/Dβ1/Dβ2 vs. Dα1/Dα2/ Dβ1/Dβ2 | 0.76 | -0.245 to 1.76 | ns | 0.3094 |
| Dα1/Dβ1/Dβ2 vs. Dα2/Dα3/ Dβ1/Dβ2 | -0.22 | -1.22 to 0.785 | ns | 0.9998 |
| Dα1/Dβ1/Dβ2 vs. Dα1/Dα2/Da3/ Dβ1/Dβ2 | 0.68 | -0.325 to 1.68 | ns | 0.4744 |
| Dα3/Dβ1/Dβ2 vs. Dα1/Dα3/ Dβ1/Dβ2 | 0.32 | -0.685 to 1.32 | ns | 0.9936 |
| Dα3/Dβ1/Dβ2 vs. Dα1/Dα2/ Dβ1/Dβ2 | 1.22 | 0.215 to 2.22 | ** | 0.0064 |
| Dα3/Dβ1/Dβ2 vs. Dα2/Dα3/ Dβ1/Dβ2 | 0.24 | -0.765 to 1.24 | ns | 0.9995 |
| Dα3/Dβ1/Dβ2 vs. Dα1/Dα2/Dα3/Dβ1/Dβ2 | 1.14 | 0.135 to 2.14 | * | 0.0142 |
| Dα1/Dα3/Dβ1/Dβ2 vs. Dα1/Dα2/Dβ1/Dβ2 | 0.90 | -0.105 to 1.90 | ns | 0.1184 |
| Dα1/Dα3/Dβ1/Dβ2 vs. Dα2/Dα3/Dβ1/Dβ2 | -0.08 | -1.08 to 0.925 | ns | >0.9999 |
| Dα1/Dα3/Dβ1/Dβ2 vs. Dα1/Dα2/Da3/Dβ1/Dβ2 | 0.82 | -0.185 to 1.82 | ns | 0.2113 |
| Dα1/Dα2/Dβ1/Dβ2 vs. Dα2/Dα3/Dβ1/Dβ2 | -0.98 | -1.98 to 0.0245 | ns | 0.0618 |
| Dα1/Dα2/Dβ1/Dβ2 vs. Dα1/Dα2/Dα3/Dβ1/Dβ2 | -0.08 | -1.08 to 0.925 | ns | >0.9999 |
| Dα2/Dα3/Dβ1/Dβ2 vs. Dα1/Dα2/Dα3/Dβ1/Dβ2 | 0.90 | -0.105 to 1.90 | ns | 0.1184 |

**^†^**One-way ANOVA, Tukey test, *, *P* < 0.05; **, *P* < 0.01; *****, *P* < 0.001; ****, *P* < 0.0001; ns, not significant.

Table E. One-way ANOVA of the I_max_ values of thiacloprid for *D. melanogaster* nAChRs

| **nAChRs compared** | **Difference** | **95% CI of difference** | **Summary^†^** | **Adjusted *P* Value** |
| --- | --- | --- | --- | --- |
| Dα1/Dβ1 vs. Dα3/Dβ1 | 0.075 | 0.0394 to 0.111 | **** | <0.0001 |
| Dα1/Dβ1 vs. Dα1/Dα3/Dβ1 | 0.044 | 0.00840 to 0.0796 | ** | 0.0051 |
| Dα1/Dβ1 vs. Dα1/Dα2/Dβ1 | 0.066 | 0.0304 to 0.102 | **** | <0.0001 |
| Dα1/Dβ1 vs. Dα2/Dα3/Dβ1 | 0.046 | 0.0104 to 0.0816 | ** | 0.0028 |
| Dα1/Dβ1 vs. Dα1/Dα2/Dα3/Dβ1 | 0.044 | 0.00840 to 0.0796 | ** | 0.0051 |
| Dα1/Dβ1 vs. Dα1/Dβ1/Dβ2 | 0.048 | 0.0124 to 0.0836 | ** | 0.0015 |
| Dα1/Dβ1 vs. Dα3/Dβ1/Dβ2 | 0.069 | 0.0334 to 0.105 | **** | <0.0001 |
| Dα1/Dβ1 vs. Dα1/Dα3/Dβ1/Dβ2 | -0.016 | -0.0516 to 0.0196 | ns | 0.9203 |
| Dα1/Dβ1 vs. Dα1/Dα2/Dβ1/Dβ2 | 0.017 | -0.0186 to 0.0526 | ns | 0.8853 |
| Dα1/Dβ1 vs. Dα2/Dα3/Dβ1/Dβ2 | 0.074 | 0.0384 to 0.110 | **** | <0.0001 |
| Dα1/Dβ1 vs. Dα1/Dα2/Dα3/Dβ1/Dβ2 | 0.032 | -0.00360 to 0.0676 | ns | 0.1158 |
| Dα3/Dβ1 vs. Dα1/Dα3/Dβ1 | -0.031 | -0.0666 to 0.00460 | ns | 0.1434 |
| Dα3/Dβ1 vs. Dα1/Dα2/Dβ1 | -0.009 | -0.0446 to 0.0266 | ns | 0.9992 |
| Dα3/Dβ1 vs. Dα2/Dα3/Dβ1 | -0.029 | -0.0646 to 0.00660 | ns | 0.2138 |
| Dα3/Dβ1 vs. Dα1/Dα2/Dα3/Dβ1 | -0.031 | -0.0666 to 0.00460 | ns | 0.1434 |
| Dα3/Dβ1 vs. Dα1/Dβ1/Dβ2 | -0.027 | -0.0626 to 0.00860 | ns | 0.306 |
| Dα3/Dβ1 vs. Dα3/Dβ1/Dβ2 | -0.006 | -0.0416 to 0.0296 | ns | >0.9999 |
| Dα3/Dβ1 vs. Dα1/Dα3/Dβ1/Dβ2 | -0.091 | -0.127 to -0.0554 | **** | <0.0001 |
| Dα3/Dβ1 vs. Dα1/Dα2/Dβ1/Dβ2 | -0.058 | -0.0936 to -0.0224 | **** | <0.0001 |
| Dα3/Dβ1 vs. Dα2/Dα3/Dβ1/Dβ2 | -0.001 | -0.0366 to 0.0346 | ns | >0.9999 |
| Dα3/Dβ1 vs. Dα1/Dα2/Dα3/Dβ1/Dβ2 | -0.043 | -0.0786 to -0.00740 | ** | 0.0068 |
| Dα1/Dα3/Dβ1 vs. Dα1/Dα2/Dβ1 | 0.022 | -0.0136 to 0.0576 | ns | 0.6104 |
| Dα1/Dα3/Dβ1 vs. Dα2/Dα3/Dβ1 | 0.002 | -0.0336 to 0.0376 | ns | >0.9999 |
| Dα1/Dα3/Dβ1 vs. Dα1/Dα2/Dα3/Dβ1 | 0.000 | -0.0356 to 0.0356 | ns | >0.9999 |
| Dα1/Dα3/Dβ1 vs. Dα1/Dβ1/Dβ2 | 0.004 | -0.0316 to 0.0396 | ns | >0.9999 |
| Dα1/Dα3/Dβ1 vs. Dα3/Dβ1/Dβ2 | 0.025 | -0.0106 to 0.0606 | ns | 0.4186 |
| Dα1/Dα3/Dβ1 vs. Dα1/Dα3/Dβ1/Dβ2 | -0.060 | -0.0956 to -0.0244 | **** | <0.0001 |
| Dα1/Dα3/Dβ1 vs. Dα1/Dα2/Dβ1/Dβ2 | -0.027 | -0.0626 to 0.00860 | ns | 0.306 |
| Dα1/Dα3/Dβ1 vs. Dα2/Dα3/Dβ1/Dβ2 | 0.030 | -0.00560 to 0.0656 | ns | 0.176 |
| Dα1/Dα3/Dβ1 vs. Dα1/Dα2/Dα3/Dβ1/Dβ2 | -0.012 | -0.0476 to 0.0236 | ns | 0.9898 |
| Dα1/Dα2/Dβ1 vs. Dα2/Dα3/Db1 | -0.020 | -0.0556 to 0.0156 | ns | 0.7358 |
| Dα1/Dα2/Dβ1 vs. Dα1/Dα2/Dα3/Db1 | -0.022 | -0.0576 to 0.0136 | ns | 0.6104 |
| Dα1/Dα2/Dβ1 vs. Dα1/Dβ1/Dβ2 | -0.018 | -0.0536 to 0.0176 | ns | 0.8424 |
| Dα1/Dα2/Dβ1 vs. Dα3/ Dβ1/Dβ2 | 0.003 | -0.0326 to 0.0386 | ns | >0.9999 |
| Dα1/Dα2/Dβ1 vs. Dα1/Dα3/Dβ1/Dβ2 | -0.082 | -0.118 to -0.0464 | **** | <0.0001 |
| Dα1/Dα2/Dβ1 vs. Dα1/Dα2/Dβ1/Dβ2 | -0.049 | -0.0846 to -0.0134 | ** | 0.0011 |
| Dα1/Dα2/Dβ1 vs. Dα2/Dα3/Dβ1/Dβ2 | 0.008 | -0.0276 to 0.0436 | ns | 0.9997 |
| Dα1/Dα2/Dβ1 vs. Dα1/Dα2/Dα3/Dβ1/Dβ2 | -0.034 | -0.0696 to 0.00160 | ns | 0.0736 |
| Dα2/Dα3/Dβ1 vs. Dα1/Dα2/Dα3/Dβ1 | -0.002 | -0.0376 to 0.0336 | ns | >0.9999 |
| Dα2/Dα3/Dβ1 vs. Dα1/Dβ1/Dβ2 | 0.002 | -0.0336 to 0.0376 | ns | >0.9999 |
| Dα2/Dα3/Dβ1 vs. Dα3/ Dβ1/Dβ2 | 0.023 | -0.0126 to 0.0586 | ns | 0.5452 |
| Dα2/Dα3/Dβ1 vs. Dα1/Dα3/Dβ1/Dβ2 | -0.062 | -0.0976 to -0.0264 | **** | <0.0001 |
| Dα2/Dα3/Dβ1 vs. Dα1/Dα2/Dβ1/Dβ2 | -0.029 | -0.0646 to 0.00660 | ns | 0.2138 |
| Dα2/Dα3/Dβ1 vs. Dα2/Dα3/Dβ1/Dβ2 | 0.028 | -0.00760 to 0.0636 | ns | 0.2572 |
| Dα2/Dα3/Dβ1 vs. Dα1/Dα2/Da3/Dβ1/Dβ2 | -0.014 | -0.0496 to 0.0216 | ns | 0.9673 |
| Dα1/Dα2/Dα3/Dβ1 vs. Dα1/Dβ1/Dβ2 | 0.004 | -0.0316 to 0.0396 | ns | >0.9999 |
| Dα1/Dα2/Dα3/Dβ1 vs. Dα3/Dβ1/Dβ2 | 0.025 | -0.0106 to 0.0606 | ns | 0.4186 |
| Dα1/Dα2/Dα3/Dβ1 vs. Dα1/Dα3/Dβ1/Dβ2 | -0.060 | -0.0956 to -0.0244 | **** | <0.0001 |
| Dα1/Dα2/Dα3/Dβ1 vs. Dα1/Dα2/Dβ1/Dβ2 | -0.027 | -0.0626 to 0.00860 | ns | 0.306 |
| Dα1/Dα2/Dα3/Dβ1 vs. Dα2/Dα3/Dβ1/Dβ2 | 0.030 | -0.00560 to 0.0656 | ns | 0.176 |
| Dα1/Dα2/Dα3/Dβ1 vs. Dα1/Dα2/Dα3/Dβ1/Dβ2 | -0.012 | -0.0476 to 0.0236 | ns | 0.9898 |
| Dα1/Dβ1/Dβ2 vs. Dα3/Dβ1/Dβ2 | 0.021 | -0.0146 to 0.0566 | ns | 0.6746 |
| Dα1/Dβ1/Dβ2 vs. Dα1/Dα3/ Dβ1/Dβ2 | -0.064 | -0.0996 to -0.0284 | **** | <0.0001 |
| Dα1/Dβ1/Dβ2 vs. Dα1/Dα2/ Dβ1/Dβ2 | -0.031 | -0.0666 to 0.00460 | ns | 0.1434 |
| Dα1/Dβ1/Dβ2 vs. Dα2/Dα3/ Dβ1/Dβ2 | 0.026 | -0.00960 to 0.0616 | ns | 0.36 |
| Dα1/Dβ1/Dβ2 vs. Dα1/Dα2/Da3/ Dβ1/Dβ2 | -0.016 | -0.0516 to 0.0196 | ns | 0.9203 |
| Dα3/Dβ1/Dβ2 vs. Dα1/Dα3/ Dβ1/Dβ2 | -0.085 | -0.121 to -0.0494 | **** | <0.0001 |
| Dα3/Dβ1/Dβ2 vs. Dα1/Dα2/ Dβ1/Dβ2 | -0.052 | -0.0876 to -0.0164 | *** | 0.0004 |
| Dα3/Dβ1/Dβ2 vs. Dα2/Dα3/ Dβ1/Dβ2 | 0.005 | -0.0306 to 0.0406 | ns | >0.9999 |
| Dα3/Dβ1/Dβ2 vs. Dα1/Dα2/Dα3/Dβ1/Dβ2 | -0.037 | -0.0726 to -0.00140 | * | 0.0352 |
| Dα1/Dα3/Dβ1/Dβ2 vs. Dα1/Dα2/Dβ1/Dβ2 | 0.033 | -0.00260 to 0.0686 | ns | 0.0927 |
| Dα1/Dα3/Dβ1/Dβ2 vs. Dα2/Dα3/Dβ1/Dβ2 | 0.090 | 0.0544 to 0.126 | **** | <0.0001 |
| Dα1/Dα3/Dβ1/Dβ2 vs. Dα1/Dα2/Da3/Dβ1/Dβ2 | 0.048 | 0.0124 to 0.0836 | ** | 0.0015 |
| Dα1/Dα2/Dβ1/Dβ2 vs. Dα2/Dα3/Dβ1/Dβ2 | 0.057 | 0.0214 to 0.0926 | **** | <0.0001 |
| Dα1/Dα2/Dβ1/Dβ2 vs. Dα1/Dα2/Dα3/Dβ1/Dβ2 | 0.015 | -0.0206 to 0.0506 | ns | 0.9474 |
| Dα2/Dα3/Dβ1/Dβ2 vs. Dα1/Dα2/Dα3/Dβ1/Dβ2 | -0.042 | -0.0776 to -0.00640 | ** | 0.0091 |

**^†^**One-way ANOVA, Tukey test, *, *P* < 0.05; **, *P* < 0.01; *****, *P* < 0.001; ****, *P* < 0.0001; ns, not significant.

Table F. One-way ANOVA of the pEC_50_ values of clothianidin for *D. melanogaster* nAChRs

| **nAChRs compared** | **Difference** | **95% CI of difference** | **Summary^†^** | **Adjusted *P* Value** |
| --- | --- | --- | --- | --- |
| Dα1/Dβ1 vs. Dα3/Dβ1 | -0.41 | -0.817 to -0.00323 | * | 0.0466 |
| Dα1/Dβ1 vs. Dα1/Dα3/Dβ1 | -0.07 | -0.477 to 0.337 | ns | >0.9999 |
| Dα1/Dβ1 vs. Dα1/Dα2/Dβ1 | 2.06 | 1.65 to 2.47 | **** | <0.0001 |
| Dα1/Dβ1 vs. Dα2/Dα3/Dβ1 | 0.32 | -0.0868 to 0.727 | ns | 0.2569 |
| Dα1/Dβ1 vs. Dα1/Dα2/Dα3/Dβ1 | 0.58 | 0.173 to 0.987 | *** | 0.0006 |
| Dα1/Dβ1 vs. Dα1/Dβ1/Dβ2 | 0.49 | 0.0832 to 0.897 | ** | 0.007 |
| Dα1/Dβ1 vs. Dα3/Dβ1/Dβ2 | -0.33 | -0.737 to 0.0768 | ns | 0.2186 |
| Dα1/Dβ1 vs. Dα1/Dα3/Dβ1/Dβ2 | -0.27 | -0.677 to 0.137 | ns | 0.5043 |
| Dα1/Dβ1 vs. Dα1/Dα2/Dβ1/Dβ2 | 0.82 | 0.413 to 1.23 | **** | <0.0001 |
| Dα1/Dβ1 vs. Dα2/Dα3/Dβ1/Dβ2 | -0.16 | -0.567 to 0.247 | ns | 0.9673 |
| Dα1/Dβ1 vs. Dα1/Dα2/Dα3/Dβ1/Dβ2 | 0.54 | 0.133 to 0.947 | ** | 0.0019 |
| Dα3/Dβ1 vs. Dα1/Dα3/Dβ1 | 0.34 | -0.0668 to 0.747 | ns | 0.1846 |
| Dα3/Dβ1 vs. Dα1/Dα2/Dβ1 | 2.47 | 2.06 to 2.88 | **** | <0.0001 |
| Dα3/Dβ1 vs. Dα2/Dα3/Dβ1 | 0.73 | 0.323 to 1.14 | **** | <0.0001 |
| Dα3/Dβ1 vs. Dα1/Dα2/Dα3/Dβ1 | 0.99 | 0.583 to 1.40 | **** | <0.0001 |
| Dα3/Dβ1 vs. Dα1/Dβ1/Dβ2 | 0.90 | 0.493 to 1.31 | **** | <0.0001 |
| Dα3/Dβ1 vs. Dα3/Dβ1/Dβ2 | 0.08 | -0.327 to 0.487 | ns | >0.9999 |
| Dα3/Dβ1 vs. Dα1/Dα3/Dβ1/Dβ2 | 0.14 | -0.267 to 0.547 | ns | 0.988 |
| Dα3/Dβ1 vs. Dα1/Dα2/Dβ1/Dβ2 | 1.23 | 0.823 to 1.64 | **** | <0.0001 |
| Dα3/Dβ1 vs. Dα2/Dα3/Dβ1/Dβ2 | 0.25 | -0.157 to 0.657 | ns | 0.6182 |
| Dα3/Dβ1 vs. Dα1/Dα2/Dα3/Dβ1/Dβ2 | 0.95 | 0.543 to 1.36 | **** | <0.0001 |
| Dα1/Dα3/Dβ1 vs. Dα1/Dα2/Dβ1 | 2.13 | 1.72 to 2.54 | **** | <0.0001 |
| Dα1/Dα3/Dβ1 vs. Dα2/Dα3/Dβ1 | 0.39 | -0.0168 to 0.797 | ns | 0.0713 |
| Dα1/Dα3/Dβ1 vs. Dα1/Dα2/Dα3/Dβ1 | 0.65 | 0.243 to 1.06 | **** | <0.0001 |
| Dα1/Dα3/Dβ1 vs. Dα1/Dβ1/Dβ2 | 0.56 | 0.153 to 0.967 | ** | 0.0011 |
| Dα1/Dα3/Dβ1 vs. Dα3/Dβ1/Dβ2 | -0.26 | -0.667 to 0.147 | ns | 0.5611 |
| Dα1/Dα3/Dβ1 vs. Dα1/Dα3/Dβ1/Dβ2 | -0.20 | -0.607 to 0.207 | ns | 0.8646 |
| Dα1/Dα3/Dβ1 vs. Dα1/Dα2/Dβ1/Dβ2 | 0.89 | 0.483 to 1.30 | **** | <0.0001 |
| Dα1/Dα3/Dβ1 vs. Dα2/Dα3/Dβ1/Dβ2 | -0.09 | -0.497 to 0.317 | ns | 0.9998 |
| Dα1/Dα3/Dβ1 vs. Dα1/Dα2/Dα3/Dβ1/Dβ2 | 0.61 | 0.203 to 1.02 | *** | 0.0003 |
| Dα1/Dα2/Dβ1 vs. Dα2/Dα3/Db1 | -1.74 | -2.15 to -1.33 | **** | <0.0001 |
| Dα1/Dα2/Dβ1 vs. Dα1/Dα2/Dα3/Db1 | -1.48 | -1.89 to -1.07 | **** | <0.0001 |
| Dα1/Dα2/Dβ1 vs. Dα1/Dβ1/Dβ2 | -1.57 | -1.98 to -1.16 | **** | <0.0001 |
| Dα1/Dα2/Dβ1 vs. Dα3/ Dβ1/Dβ2 | -2.39 | -2.80 to -1.98 | **** | <0.0001 |
| Dα1/Dα2/Dβ1 vs. Dα1/Dα3/Dβ1/Dβ2 | -2.33 | -2.74 to -1.92 | **** | <0.0001 |
| Dα1/Dα2/Dβ1 vs. Dα1/Dα2/Dβ1/Dβ2 | -1.24 | -1.65 to -0.833 | **** | <0.0001 |
| Dα1/Dα2/Dβ1 vs. Dα2/Dα3/Dβ1/Dβ2 | -2.22 | -2.63 to -1.81 | **** | <0.0001 |
| Dα1/Dα2/Dβ1 vs. Dα1/Dα2/Dα3/Dβ1/Dβ2 | -1.52 | -1.93 to -1.11 | **** | <0.0001 |
| Dα2/Dα3/Dβ1 vs. Dα1/Dα2/Dα3/Dβ1 | 0.26 | -0.147 to 0.667 | ns | 0.5611 |
| Dα2/Dα3/Dβ1 vs. Dα1/Dβ1/Dβ2 | 0.17 | -0.237 to 0.577 | ns | 0.9502 |
| Dα2/Dα3/Dβ1 vs. Dα3/ Dβ1/Dβ2 | -0.65 | -1.06 to -0.243 | **** | <0.0001 |
| Dα2/Dα3/Dβ1 vs. Dα1/Dα3/Dβ1/Dβ2 | -0.59 | -0.997 to -0.183 | *** | 0.0005 |
| Dα2/Dα3/Dβ1 vs. Dα1/Dα2/Dβ1/Dβ2 | 0.50 | 0.0932 to 0.907 | ** | 0.0055 |
| Dα2/Dα3/Dβ1 vs. Dα2/Dα3/Dβ1/Dβ2 | -0.48 | -0.887 to -0.0732 | ** | 0.009 |
| Dα2/Dα3/Dβ1 vs. Dα1/Dα2/Da3/Dβ1/Dβ2 | 0.22 | -0.187 to 0.627 | ns | 0.7784 |
| Dα1/Dα2/Dα3/Dβ1 vs. Dα1/Dβ1/Dβ2 | -0.09 | -0.497 to 0.317 | ns | 0.9998 |
| Dα1/Dα2/Dα3/Dβ1 vs. Dα3/Dβ1/Dβ2 | -0.91 | -1.32 to -0.503 | **** | <0.0001 |
| Dα1/Dα2/Dα3/Dβ1 vs. Dα1/Dα3/Dβ1/Dβ2 | -0.85 | -1.26 to -0.443 | **** | <0.0001 |
| Dα1/Dα2/Dα3/Dβ1 vs. Dα1/Dα2/Dβ1/Dβ2 | 0.24 | -0.167 to 0.647 | ns | 0.6742 |
| Dα1/Dα2/Dα3/Dβ1 vs. Dα2/Dα3/Dβ1/Dβ2 | -0.74 | -1.15 to -0.333 | **** | <0.0001 |
| Dα1/Dα2/Dα3/Dβ1 vs. Dα1/Dα2/Dα3/Dβ1/Dβ2 | -0.04 | -0.447 to 0.367 | ns | >0.9999 |
| Dα1/Dβ1/Dβ2 vs. Dα3/Dβ1/Dβ2 | -0.82 | -1.23 to -0.413 | **** | <0.0001 |
| Dα1/Dβ1/Dβ2 vs. Dα1/Dα3/ Dβ1/Dβ2 | -0.76 | -1.17 to -0.353 | **** | <0.0001 |
| Dα1/Dβ1/Dβ2 vs. Dα1/Dα2/ Dβ1/Dβ2 | 0.33 | -0.0768 to 0.737 | ns | 0.2186 |
| Dα1/Dβ1/Dβ2 vs. Dα2/Dα3/ Dβ1/Dβ2 | -0.65 | -1.06 to -0.243 | **** | <0.0001 |
| Dα1/Dβ1/Dβ2 vs. Dα1/Dα2/Da3/ Dβ1/Dβ2 | 0.05 | -0.357 to 0.457 | ns | >0.9999 |
| Dα3/Dβ1/Dβ2 vs. Dα1/Dα3/ Dβ1/Dβ2 | 0.06 | -0.347 to 0.467 | ns | >0.9999 |
| Dα3/Dβ1/Dβ2 vs. Dα1/Dα2/ Dβ1/Dβ2 | 1.15 | 0.743 to 1.56 | **** | <0.0001 |
| Dα3/Dβ1/Dβ2 vs. Dα2/Dα3/ Dβ1/Dβ2 | 0.17 | -0.237 to 0.577 | ns | 0.9502 |
| Dα3/Dβ1/Dβ2 vs. Dα1/Dα2/Dα3/Dβ1/Dβ2 | 0.87 | 0.463 to 1.28 | **** | <0.0001 |
| Dα1/Dα3/Dβ1/Dβ2 vs. Dα1/Dα2/Dβ1/Dβ2 | 1.09 | 0.683 to 1.50 | **** | <0.0001 |
| Dα1/Dα3/Dβ1/Dβ2 vs. Dα2/Dα3/Dβ1/Dβ2 | 0.11 | -0.297 to 0.517 | ns | 0.9984 |
| Dα1/Dα3/Dβ1/Dβ2 vs. Dα1/Dα2/Da3/Dβ1/Dβ2 | 0.81 | 0.403 to 1.22 | **** | <0.0001 |
| Dα1/Dα2/Dβ1/Dβ2 vs. Dα2/Dα3/Dβ1/Dβ2 | -0.98 | -1.39 to -0.573 | **** | <0.0001 |
| Dα1/Dα2/Dβ1/Dβ2 vs. Dα1/Dα2/Dα3/Dβ1/Dβ2 | -0.28 | -0.687 to 0.127 | ns | 0.4489 |
| Dα2/Dα3/Dβ1/Dβ2 vs. Dα1/Dα2/Dα3/Dβ1/Dβ2 | 0.70 | 0.293 to 1.11 | **** | <0.0001 |

**^†^**One-way ANOVA, Tukey test, *, *P* < 0.05; **, *P* < 0.01; *****, *P* < 0.001; ****, *P* < 0.0001; ns, not significant.

Table G. One-way ANOVA of the I_max_ values of clothianidin for *D. melanogaster* nAChRs

| **nAChRs compared** | **Difference** | **95% CI of difference** | **Summary^†^** | **Adjusted *P* Value** |
| --- | --- | --- | --- | --- |
| Dα1/Dβ1 vs. Dα3/Dβ1 | -0.183 | -0.332 to -0.0335 | ** | 0.0057 |
| Dα1/Dβ1 vs. Dα1/Dα3/Dβ1 | -0.119 | -0.268 to 0.0305 | ns | 0.2419 |
| Dα1/Dβ1 vs. Dα1/Dα2/Dβ1 | -0.191 | -0.340 to -0.0415 | ** | 0.0033 |
| Dα1/Dβ1 vs. Dα2/Dα3/Dβ1 | -0.374 | -0.523 to -0.225 | **** | <0.0001 |
| Dα1/Dβ1 vs. Dα1/Dα2/Dα3/Dβ1 | -0.309 | -0.458 to -0.160 | **** | <0.0001 |
| Dα1/Dβ1 vs. Dα1/Dβ1/Dβ2 | 0.148 | -0.00150 to 0.297 | ns | 0.0546 |
| Dα1/Dβ1 vs. Dα3/Dβ1/Dβ2 | 0.016 | -0.133 to 0.165 | ns | >0.9999 |
| Dα1/Dβ1 vs. Dα1/Dα3/Dβ1/Dβ2 | -0.176 | -0.325 to -0.0265 | ** | 0.0093 |
| Dα1/Dβ1 vs. Dα1/Dα2/Dβ1/Dβ2 | -0.292 | -0.441 to -0.143 | **** | <0.0001 |
| Dα1/Dβ1 vs. Dα2/Dα3/Dβ1/Dβ2 | -0.126 | -0.275 to 0.0235 | ns | 0.1757 |
| Dα1/Dβ1 vs. Dα1/Dα2/Dα3/Dβ1/Dβ2 | -0.304 | -0.453 to -0.155 | **** | <0.0001 |
| Dα3/Dβ1 vs. Dα1/Dα3/Dβ1 | 0.064 | -0.0855 to 0.213 | ns | 0.9416 |
| Dα3/Dβ1 vs. Dα1/Dα2/Dβ1 | -0.008 | -0.157 to 0.141 | ns | >0.9999 |
| Dα3/Dβ1 vs. Dα2/Dα3/Dβ1 | -0.191 | -0.340 to -0.0415 | ** | 0.0033 |
| Dα3/Dβ1 vs. Dα1/Dα2/Dα3/Dβ1 | -0.126 | -0.275 to 0.0235 | ns | 0.1757 |
| Dα3/Dβ1 vs. Dα1/Dβ1/Dβ2 | 0.331 | 0.182 to 0.480 | **** | <0.0001 |
| Dα3/Dβ1 vs. Dα3/Dβ1/Dβ2 | 0.199 | 0.0495 to 0.348 | ** | 0.0018 |
| Dα3/Dβ1 vs. Dα1/Dα3/Dβ1/Dβ2 | 0.007 | -0.142 to 0.156 | ns | >0.9999 |
| Dα3/Dβ1 vs. Dα1/Dα2/Dβ1/Dβ2 | -0.109 | -0.258 to 0.0405 | ns | 0.3624 |
| Dα3/Dβ1 vs. Dα2/Dα3/Dβ1/Dβ2 | 0.057 | -0.0925 to 0.206 | ns | 0.9739 |
| Dα3/Dβ1 vs. Dα1/Dα2/Dα3/Dβ1/Dβ2 | -0.121 | -0.270 to 0.0285 | ns | 0.2214 |
| Dα1/Dα3/Dβ1 vs. Dα1/Dα2/Dβ1 | -0.072 | -0.221 to 0.0775 | ns | 0.8795 |
| Dα1/Dα3/Dβ1 vs. Dα2/Dα3/Dβ1 | -0.255 | -0.404 to -0.106 | **** | <0.0001 |
| Dα1/Dα3/Dβ1 vs. Dα1/Dα2/Dα3/Dβ1 | -0.190 | -0.339 to -0.0405 | ** | 0.0035 |
| Dα1/Dα3/Dβ1 vs. Dα1/Dβ1/Dβ2 | 0.267 | 0.118 to 0.416 | **** | <0.0001 |
| Dα1/Dα3/Dβ1 vs. Dα3/Dβ1/Dβ2 | 0.135 | -0.0145 to 0.284 | ns | 0.112 |
| Dα1/Dα3/Dβ1 vs. Dα1/Dα3/Dβ1/Dβ2 | -0.057 | -0.206 to 0.0925 | ns | 0.9739 |
| Dα1/Dα3/Dβ1 vs. Dα1/Dα2/Dβ1/Dβ2 | -0.173 | -0.322 to -0.0235 | * | 0.0114 |
| Dα1/Dα3/Dβ1 vs. Dα2/Dα3/Dβ1/Dβ2 | -0.007 | -0.156 to 0.142 | ns | >0.9999 |
| Dα1/Dα3/Dβ1 vs. Dα1/Dα2/Dα3/Dβ1/Dβ2 | -0.185 | -0.334 to -0.0355 | ** | 0.005 |
| Dα1/Dα2/Dβ1 vs. Dα2/Dα3/Db1 | -0.183 | -0.332 to -0.0335 | ** | 0.0057 |
| Dα1/Dα2/Dβ1 vs. Dα1/Dα2/Dα3/Db1 | -0.118 | -0.267 to 0.0315 | ns | 0.2526 |
| Dα1/Dα2/Dβ1 vs. Dα1/Dβ1/Dβ2 | 0.339 | 0.190 to 0.488 | **** | <0.0001 |
| Dα1/Dα2/Dβ1 vs. Dα3/ Dβ1/Dβ2 | 0.207 | 0.0575 to 0.356 | ** | 0.001 |
| Dα1/Dα2/Dβ1 vs. Dα1/Dα3/Dβ1/Dβ2 | 0.015 | -0.134 to 0.164 | ns | >0.9999 |
| Dα1/Dα2/Dβ1 vs. Dα1/Dα2/Dβ1/Dβ2 | -0.101 | -0.250 to 0.0485 | ns | 0.4774 |
| Dα1/Dα2/Dβ1 vs. Dα2/Dα3/Dβ1/Dβ2 | 0.065 | -0.0845 to 0.214 | ns | 0.9354 |
| Dα1/Dα2/Dβ1 vs. Dα1/Dα2/Dα3/Dβ1/Dβ2 | -0.113 | -0.262 to 0.0365 | ns | 0.3107 |
| Dα2/Dα3/Dβ1 vs. Dα1/Dα2/Dα3/Dβ1 | 0.065 | -0.0845 to 0.214 | ns | 0.9354 |
| Dα2/Dα3/Dβ1 vs. Dα1/Dβ1/Dβ2 | 0.522 | 0.373 to 0.671 | **** | <0.0001 |
| Dα2/Dα3/Dβ1 vs. Dα3/ Dβ1/Dβ2 | 0.390 | 0.241 to 0.539 | **** | <0.0001 |
| Dα2/Dα3/Dβ1 vs. Dα1/Dα3/Dβ1/Dβ2 | 0.198 | 0.0485 to 0.347 | ** | 0.002 |
| Dα2/Dα3/Dβ1 vs. Dα1/Dα2/Dβ1/Dβ2 | 0.082 | -0.0675 to 0.231 | ns | 0.7631 |
| Dα2/Dα3/Dβ1 vs. Dα2/Dα3/Dβ1/Dβ2 | 0.248 | 0.0985 to 0.397 | **** | <0.0001 |
| Dα2/Dα3/Dβ1 vs. Dα1/Dα2/Da3/Dβ1/Dβ2 | 0.070 | -0.0795 to 0.219 | ns | 0.8977 |
| Dα1/Dα2/Dα3/Dβ1 vs. Dα1/Dβ1/Dβ2 | 0.457 | 0.308 to 0.606 | **** | <0.0001 |
| Dα1/Dα2/Dα3/Dβ1 vs. Dα3/Dβ1/Dβ2 | 0.325 | 0.176 to 0.474 | **** | <0.0001 |
| Dα1/Dα2/Dα3/Dβ1 vs. Dα1/Dα3/Dβ1/Dβ2 | 0.133 | -0.0165 to 0.282 | ns | 0.1243 |
| Dα1/Dα2/Dα3/Dβ1 vs. Dα1/Dα2/Dβ1/Dβ2 | 0.017 | -0.132 to 0.166 | ns | >0.9999 |
| Dα1/Dα2/Dα3/Dβ1 vs. Dα2/Dα3/Dβ1/Dβ2 | 0.183 | 0.0335 to 0.332 | ** | 0.0057 |
| Dα1/Dα2/Dα3/Dβ1 vs. Dα1/Dα2/Dα3/Dβ1/Dβ2 | 0.005 | -0.144 to 0.154 | ns | >0.9999 |
| Dα1/Dβ1/Dβ2 vs. Dα3/Dβ1/Dβ2 | -0.132 | -0.281 to 0.0175 | ns | 0.1308 |
| Dα1/Dβ1/Dβ2 vs. Dα1/Dα3/ Dβ1/Dβ2 | -0.324 | -0.473 to -0.175 | **** | <0.0001 |
| Dα1/Dβ1/Dβ2 vs. Dα1/Dα2/ Dβ1/Dβ2 | -0.440 | -0.589 to -0.291 | **** | <0.0001 |
| Dα1/Dβ1/Dβ2 vs. Dα2/Dα3/ Dβ1/Dβ2 | -0.274 | -0.423 to -0.125 | **** | <0.0001 |
| Dα1/Dβ1/Dβ2 vs. Dα1/Dα2/Da3/ Dβ1/Dβ2 | -0.452 | -0.601 to -0.303 | **** | <0.0001 |
| Dα3/Dβ1/Dβ2 vs. Dα1/Dα3/ Dβ1/Dβ2 | -0.192 | -0.341 to -0.0425 | ** | 0.003 |
| Dα3/Dβ1/Dβ2 vs. Dα1/Dα2/ Dβ1/Dβ2 | -0.308 | -0.457 to -0.159 | **** | <0.0001 |
| Dα3/Dβ1/Dβ2 vs. Dα2/Dα3/ Dβ1/Dβ2 | -0.142 | -0.291 to 0.00750 | ns | 0.0768 |
| Dα3/Dβ1/Dβ2 vs. Dα1/Dα2/Dα3/Dβ1/Dβ2 | -0.320 | -0.469 to -0.171 | **** | <0.0001 |
| Dα1/Dα3/Dβ1/Dβ2 vs. Dα1/Dα2/Dβ1/Dβ2 | -0.116 | -0.265 to 0.0335 | ns | 0.2749 |
| Dα1/Dα3/Dβ1/Dβ2 vs. Dα2/Dα3/Dβ1/Dβ2 | 0.050 | -0.0995 to 0.199 | ns | 0.9904 |
| Dα1/Dα3/Dβ1/Dβ2 vs. Dα1/Dα2/Da3/ Dβ1/Dβ2 | -0.128 | -0.277 to 0.0215 | ns | 0.1596 |
| Dα1/Dα2/Dβ1/Dβ2 vs. Dα2/Dα3/Dβ1/Dβ2 | 0.166 | 0.0165 to 0.315 | * | 0.018 |
| Dα1/Dα2/Dβ1/Dβ2 vs. Dα1/Dα2/Dα3/Dβ1/Dβ2 | -0.012 | -0.161 to 0.137 | ns | >0.9999 |
| Dα2/Dα3/Dβ1/Dβ2 vs. Dα1/Dα2/Dα3/Dβ1/Dβ2 | -0.178 | -0.327 to -0.0285 | ** | 0.0081 |

**^†^**One-way ANOVA, Tukey test, *, *P* < 0.05; **, *P* < 0.01; *****, *P* < 0.001; ****, *P* < 0.0001; ns, not significant.

**Table H.** Subunit parameters and data set for multivariate analyses

| **nAChRs** | **Subunits** | | | | | **Affinity** | **Efficacy** |
| --- | --- | --- | --- | --- | --- | --- | --- |
|  | **Dα1** | **Dα2** | **Dα3** | **Dβ1** | **Dβ2** | **pEC_50_** | **I_max_** |
| **Acetylcholine** |  |  |  |  |  |  |  |
| Dα1/Dβ1 | 1 | 0 | 0 | 1 | 0 | 5.24 | 1 |
| Dα3/Dβ1 | 0 | 0 | 1 | 1 | 0 | 6.14 | 1 |
| Dα1/Dα3/Dβ1 | 1 | 0 | 1 | 1 | 0 | 5.85 | 1 |
| Dα1/Dα2/Dβ1 | 1 | 1 | 0 | 1 | 0 | 4.14 | 1 |
| Dα2/Dα3/Dβ1 | 0 | 1 | 1 | 1 | 0 | 5.45 | 1 |
| Dα1/Dα2/Dα3/Dβ1 | 1 | 1 | 1 | 1 | 0 | 5.43 | 1 |
| Dα1/Dβ1/Dβ2 | 1 | 0 | 0 | 1 | 1 | 4.75 | 1 |
| Dα3/Dβ1/Dβ2 | 0 | 0 | 1 | 1 | 1 | 6.19 | 1 |
| Dα1/Dα3/Dβ1/Dβ2 | 1 | 0 | 1 | 1 | 1 | 6.43 | 1 |
| Dα1/Dα2/Dβ1/Dβ2 | 1 | 1 | 0 | 1 | 1 | 4.92 | 1 |
| Dα2/Dα3/Dβ1/Dβ2 | 0 | 1 | 1 | 1 | 1 | 5.99 | 1 |
| Dα1/Dα2/Dα3/Dβ1/Dβ2 | 1 | 1 | 1 | 1 | 1 | 4.49 | 1 |
|  |  |  |  |  |  |  |  |
| **Imidacloprid** |  |  |  |  |  |  |  |
| Dα1/Dβ1 | 1 | 0 | 0 | 1 | 0 | 7.16 | 0.122 |
| Dα3/Dβ1 | 0 | 0 | 1 | 1 | 0 | 8.32 | 0.032 |
| Dα1/Dα3/Dβ1 | 1 | 0 | 1 | 1 | 0 | 7.58 | 0.170 |
| Dα1/Dα2/Dβ1 | 1 | 1 | 0 | 1 | 0 | 6.71 | 0.031 |
| Dα2/Dα3/Dβ1 | 0 | 1 | 1 | 1 | 0 | 6.88 | 0.101 |
| Dα1/Dα2/Dα3/Dβ1 | 1 | 1 | 1 | 1 | 0 | 7.08 | 0.094 |
| Dα1/Dβ1/Dβ2 | 1 | 0 | 0 | 1 | 1 | 6.99 | 0.076 |
| Dα3/Dβ1/Dβ2 | 0 | 0 | 1 | 1 | 1 | 8.28 | 0.039 |
| Dα1/Dα3/Dβ1/Dβ2 | 1 | 0 | 1 | 1 | 1 | 7.52 | 0.330 |
| Dα1/Dα2/Dβ1/Dβ2 | 1 | 1 | 0 | 1 | 1 | 6.45 | 0.165 |
| Dα2/Dα3/Dβ1/Dβ2 | 0 | 1 | 1 | 1 | 1 | 7.00 | 0.125 |
| Dα1/Dα2/Dα3/Dβ1/Dβ2 | 1 | 1 | 1 | 1 | 1 | 6.47 | 0.142 |
|  |  |  |  |  |  |  |  |
| **Thiacloprid** |  |  |  |  |  |  |  |
| Dα1/Dβ1 | 1 | 0 | 0 | 1 | 0 | 7.79 | 0.091 |
| Dα3/Dβ1 | 0 | 0 | 1 | 1 | 0 | 7.80 | 0.016 |
| Dα1/Dα3/Dβ1 | 1 | 0 | 1 | 1 | 0 | 8.07 | 0.047 |
| Dα1/Dα2/Dβ1 | 1 | 1 | 0 | 1 | 0 | 6.92 | 0.025 |
| Dα2/Dα3/Dβ1 | 0 | 1 | 1 | 1 | 0 | 6.92 | 0.045 |
| Dα1/Dα2/Dα3/Dβ1 | 1 | 1 | 1 | 1 | 0 | 7.73 | 0.047 |
| Dα1/Dβ1/Dβ2 | 1 | 0 | 0 | 1 | 1 | 7.86 | 0.043 |
| Dα3/Dβ1/Dβ2 | 0 | 0 | 1 | 1 | 1 | 8.32 | 0.022 |
| Dα1/Dα3/Dβ1/Dβ2 | 1 | 0 | 1 | 1 | 1 | 8.00 | 0.107 |
| Dα1/Dα2/Dβ1/Dβ2 | 1 | 1 | 0 | 1 | 1 | 7.10 | 0.074 |
| Dα2/Dα3/Dβ1/Dβ2 | 0 | 1 | 1 | 1 | 1 | 8.08 | 0.017 |
| Dα1/Dα2/Dα3/Dβ1/Dβ2 | 1 | 1 | 1 | 1 | 1 | 7.18 | 0.059 |
|  |  |  |  |  |  |  |  |
| **Clothianidin** |  |  |  |  |  |  |  |
| Dα1/Dβ1 | 1 | 0 | 0 | 1 | 0 | 7.13 | 0.508 |
| Dα3/Dβ1 | 0 | 0 | 1 | 1 | 0 | 7.54 | 0.691 |
| Dα1/Dα3/Dβ1 | 1 | 0 | 1 | 1 | 0 | 7.20 | 0.627 |
| Dα1/Dα2/Dβ1 | 1 | 1 | 0 | 1 | 0 | 5.07 | 0.699 |
| Dα2/Dα3/Dβ1 | 0 | 1 | 1 | 1 | 0 | 6.81 | 0.882 |
| Dα1/Dα2/Dα3/Dβ1 | 1 | 1 | 1 | 1 | 0 | 6.55 | 0.817 |
| Dα1/Dβ1/Dβ2 | 1 | 0 | 0 | 1 | 1 | 6.64 | 0.360 |
| Dα3/Dβ1/Dβ2 | 0 | 0 | 1 | 1 | 1 | 7.46 | 0.492 |
| Dα1/Dα3/Dβ1/Dβ2 | 1 | 0 | 1 | 1 | 1 | 7.40 | 0.684 |
| Dα1/Dα2/Dβ1/Dβ2 | 1 | 1 | 0 | 1 | 1 | 6.31 | 0.800 |
| Dα2/Dα3/Dβ1/Dβ2 | 0 | 1 | 1 | 1 | 1 | 7.29 | 0.634 |
| Dα1/Dα2/Dα3/Dβ1/Dβ2 | 1 | 1 | 1 | 1 | 1 | 6.59 | 0.812 |

**Table I.** Mean and 95% confidence intervals of ΔpEC_50_ values obtained by lattice analysis

| **Subunits** | **Imidacloprid** | **Thiacloprid** | **Clothianidin** |
| --- | --- | --- | --- |
| Dα1 | -0.458 (-1.174 − 0.259) | -0.035 (-1.210 − 1.140) | -0.340 (-0.765 − 0.085) |
| Dα2 | -0.877 (-1.333, -0.420)* | -0.652 (-0.951 − -0.352)* | -0.792 (-1.493 − -0.090)* |
| Dα3 | 0.335 (-0.016 − 0.686) | 0.328 (-0.201 − 0.856) | 0.648 (-0.348 − 1.643) |
| Dβ2 | -0.170 (-0.433 − 0.093) | 0.218 (-0.389 − 0.826) | 0.232 (-0.386 − 0.850) |

*****The significance of the negative effect was determined by the 95% confidence interval in each parenthesis.

**Table J.** One-way ANOVA of the pEC_50_ and I_max_ values of ligands for *D. melanogaster* nAChRs containing Dα4 or Dβ3 subunit as compared with the values for Dα1/Dα2/Dα3/Dβ1/Dβ2 nAChR

| **pEC_50_ of ACh** | **Difference** | **95% CI of Difference** | **Summary^†^** | **Adjusted *P* Value** |
| --- | --- | --- | --- | --- |
| Dα1/Dα2/Dα3/Dβ1/Dβ2 vs. Dα2/Dα3/Dα4/Dβ1/Dβ2 | -1.00 | -1.19 to -0.814 | **** | <0.0001 |
| Dα1/Dα2/Dα3/Dβ1/Dβ2 vs. Dα2/Dα3/Dβ1/Dβ2/Dβ3 | -1.52 | -1.70 to -1.33 | **** | <0.0001 |
| Dα1/Dα2/Dα3/Dβ1/Dβ2 vs. Dα1/Dα3/Dα4/Dβ1/Dβ2 | -0.30 | -0.486 to -0.112 | *** | 0.0003 |
| Dα1/Dα2/Dα3/Dβ1/Dβ2 vs. Dα1/Dα3/Dβ1/Dβ2/Dβ3 | -1.34 | -1.52 to -1.15 | **** | <0.0001 |
| Dα1/Dα2/Dα3/Dβ1/Dβ2 vs. Dα1/Dα2/Dα4/Dβ1/Dβ2 | -0.15 | -0.333 to 0.0409 | ns | 0.2252 |
| Dα1/Dα2/Dα3/Dβ1/Dβ2 vs. Dα1/Dα2/Dβ1/Dβ2/Dβ3 | 0.07 | -0.113 to 0.261 | ns | >0.9999 |
| **pEC_50_ of Imidacloprid** |  |  |  |  |
| Dα1/Dα2/Dα3/Dβ1/Dβ2 vs. Dα2/Dα3/Dα4/Dβ1/Dβ2 | -0.93 | -1.71 to -0.152 | * | 0.0108 |
| Dα1/Dα2/Dα3/Dβ1/Dβ2 vs. Dα2/Dα3/Dβ1/Dβ2/Dβ3 | -1.28 | -2.06 to -0.499 | *** | 0.0002 |
| Dα1/Dα2/Dα3/Dβ1/Dβ2 vs. Dα1/Dα3/Dα4/Dβ1/Dβ2 | -0.83 | -1.61 to -0.0500 | * | 0.0308 |
| Dα1/Dα2/Dα3/Dβ1/Dβ2 vs. Dα1/Dα3/Dβ1/Dβ2/Dβ3 | -1.25 | -2.04 to -0.473 | *** | 0.0003 |
| Dα1/Dα2/Dα3/Dβ1/Dβ2 vs. Dα1/Dα2/Dα4/Dβ1/Dβ2 | -0.02 | -0.788 to 0.774 | ns | >0.9999 |
| Dα1/Dα2/Dα3/Dβ1/Dβ2 vs. Dα1/Dα2/Dβ1/Dβ2/Dβ3 | 0.21 | -0.576 to 0.986 | ns | >0.9999 |
| **I_max_ of Imidacloprid** |  |  |  |  |
| Dα1/Dα2/Dα3/Dβ1/Dβ2 vs. Dα2/Dα3/Dα4/Dβ1/Dβ2 | 0.115 | 0.0668 to 0.162 | **** | <0.0001 |
| Dα1/Dα2/Dα3/Dβ1/Dβ2 vs. Dα2/Dα3/Dβ1/Dβ2/Dβ3 | 0.121 | 0.0732 to 0.169 | **** | <0.0001 |
| Dα1/Dα2/Dα3/Dβ1/Dβ2 vs. Dα1/Dα3/Dα4/Dβ1/Dβ2 | 0.041 | -0.00649 to 0.0891 | ns | 0.1309 |
| Dα1/Dα2/Dα3/Dβ1/Dβ2 vs. Dα1/Dα3/Dβ1/Dβ2/Dβ3 | 0.002 | -0.0458 to 0.0498 | ns | >0.9999 |
| Dα1/Dα2/Dα3/Dβ1/Dβ2 vs. Dα1/Dα2/Dα4/Dβ1/Dβ2 | -0.030 | -0.0774 to 0.0182 | ns | 0.5834 |
| Dα1/Dα2/Dα3/Dβ1/Dβ2 vs. Dα1/Dα2/Dβ1/Dβ2/Dβ3 | 0.062 | 0.0138 to 0.109 | ** | 0.0048 |
| **pEC_50_ of Thiacloprid** |  |  |  |  |
| Dα1/Dα2/Dα3/Dβ1/Dβ2 vs. Dα2/Dα3/Dα4/Dβ1/Dβ2 | -0.81 | -1.65 to 0.0368 | ns | 0.0688 |
| Dα1/Dα2/Dα3/Dβ1/Dβ2 vs. Dα2/Dα3/Dβ1/Dβ2/Dβ3 | -1.13 | -1.98 to -0.290 | ** | 0.0030 |
| Dα1/Dα2/Dα3/Dβ1/Dβ2 vs. Dα1/Dα3/Dα4/Dβ1/Dβ2 | -0.78 | -1.62 to 0.0668 | ns | 0.0886 |
| Dα1/Dα2/Dα3/Dβ1/Dβ2 vs. Dα1/Dα3/Dβ1/Dβ2/Dβ3 | -1.45 | -2.29 to -0.607 | **** | <0.0001 |
| Dα1/Dα2/Dα3/Dβ1/Dβ2 vs. Dα1/Dα2/Dα4/Dβ1/Dβ2 | 0.17 | -0.669 to 1.02 | ns | >0.9999 |
| Dα1/Dα2/Dα3/Dβ1/Dβ2 vs. Dα1/Dα2/Dβ1/Dβ2/Dβ3 | -0.53 | -1.38 to 0.310 | ns | 0.5430 |
| **I_max_ of Thiacloprd** |  |  |  |  |
| Dα1/Dα2/Dα3/Dβ1/Dβ2 vs. Dα2/Dα3/Dα4/Dβ1/Dβ2 | 0.041 | 0.0154 to 0.0662 | *** | 0.0003 |
| Dα1/Dα2/Dα3/Dβ1/Dβ2 vs. Dα2/Dα3/Dβ1/Dβ2/Dβ3 | 0.049 | 0.0238 to 0.0746 | **** | <0.0001 |
| Dα1/Dα2/Dα3/Dβ1/Dβ2 vs. Dα1/Dα3/Dα4/Dβ1/Dβ2 | 0.033 | 0.00715 to 0.0579 | ** | 0.0052 |
| Dα1/Dα2/Dα3/Dβ1/Dβ2 vs. Dα1/Dα3/Dβ1/Dβ2/Dβ3 | -0.030 | -0.0556 to -0.00485 | * | 0.0111 |
| Dα1/Dα2/Dα3/Dβ1/Dβ2 vs. Dα1/Dα2/Dα4/Dβ1/Dβ2 | -0.007 | -0.0324 to 0.0183 | ns | >0.9999 |
| Dα1/Dα2/Dα3/Dβ1/Dβ2 vs. Dα1/Dα2/Dβ1/Dβ2/Dβ3 | 0.036 | 0.0106 to 0.0614 | ** | 0.0015 |
| **pEC_50_ of Clothianidin** |  |  |  |  |
| Dα1/Dα2/Dα3/Dβ1/Dβ2 vs. Dα2/Dα3/Dα4/Dβ1/Dβ2 | -0.52 | -0.856 to -0.184 | *** | 0.0005 |
| Dα1/Dα2/Dα3/Dβ1/Dβ2 vs. Dα2/Dα3/Dβ1/Dβ2/Dβ3 | -0.87 | -1.21 to -0.534 | **** | <0.0001 |
| Dα1/Dα2/Dα3/Dβ1/Dβ2 vs. Dα1/Dα3/Dα4/Dβ1/Dβ2 | -0.35 | -0.689 to -0.0166 | * | 0.0345 |
| Dα1/Dα2/Dα3/Dβ1/Dβ2 vs. Dα1/Dα3/Dβ1/Dβ2/Dβ3 | -1.01 | -1.35 to -0.678 | **** | <0.0001 |
| Dα1/Dα2/Dα3/Dβ1/Dβ2 vs. Dα1/Dα2/Dα4/Dβ1/Dβ2 | -0.13 | -0.467 to 0.205 | ns | >0.9999 |
| Dα1/Dα2/Dα3/Dβ1/Dβ2 vs. Dα1/Dα2/Dβ1/Dβ2/Dβ3 | 0.92 | 0.587 to 1.26 | **** | <0.0001 |
| **I_max_ of Clothianidin** |  |  |  |  |
| Dα1/Dα2/Dα3/Dβ1/Dβ2 vs. Dα2/Dα3/Dα4/Dβ1/Dβ2 | -0.100 | -0.217 to 0.0166 | ns | 0.1371 |
| Dα1/Dα2/Dα3/Dβ1/Dβ2 vs. Dα2/Dα3/Dβ1/Dβ2/Dβ3 | 0.117 | 0.000353 to 0.234 | * | 0.0489 |
| Dα1/Dα2/Dα3/Dβ1/Dβ2 vs. Dα1/Dα3/Dα4/Dβ1/Dβ2 | 0.388 | 0.271 to 0.504 | **** | <0.0001 |
| Dα1/Dα2/Dα3/Dβ1/Dβ2 vs. Dα1/Dα3/Dβ1/Dβ2/Dβ3 | 0.281 | 0.164 to 0.398 | **** | <0.0001 |
| Dα1/Dα2/Dα3/Dβ1/Dβ2 vs. Dα1/Dα2/Dα4/Dβ1/Dβ2 | -0.189 | -0.306 to -0.0724 | *** | 0.0002 |
| Dα1/Dα2/Dα3/Dβ1/Dβ2 vs. Dα1/Dα2/Dβ1/Dβ2/Dβ3 | -0.162 | -0.279 to -0.0456 | ** | 0.0020 |

**^†^**One-way ANOVA, Tukey test, *, *P* < 0.05; **, *P* < 0.01; *****, *P* < 0.001; ****, *P* < 0.0001; ns, not significant.

**Table K.** Primers for qRT-PCR

| **Gene** | **CG Number** | **Forward (5’-3’)** | **Reverse (5’-3’)** |
| --- | --- | --- | --- |
| *Dα1* | *CG5610* | TCATCACACGGGCAAAGTGGTGTG | TGCTTCAAGTGCCTCAAGTCCACC |
| *Dα2* | *CG6844* | TTCTAACGACCAACGTGTGGCTGG | TTCGTCATGGTGGTGACCACGTAC |
| *Dα3* | *CG2302* | AGCAGACCTGCGTCATGAAGTTCG | ATAGAACTCGGACAGATCCACGCC |
| *Dα4* | *CG12414* | TGAGGCACATGGATGAACAACAGG | TGATGTCCAAGTATGGTTCGTCGC |
| *Dα5* | *CG32975* | AAGGACGAGGATGTAGCCAACCAC | AATCGTTGTCGCTTGCGTGCACAC |
| *Dα6* | *CG4128* | AATGGCGAGTGGTACTTGCTTGCC | AATGTGAAGCCCAGTAGGGCCATC |
| *Dα7* | *CG32538* | ACAACTACAACAGCCTGGAGCGTC | CACCACCGAACTCACTCGAATTCC |
| *Dβ1* | *CG11348* | TTGGTACGACTACCAGCTGCAGTG | ATCAGCACGTTGGACTTGTAGCGC |
| *Dβ2 (Common)* | *CG6798* | ACCCTGACGGTTTGGTTAGGTCTG | TTCAACTCCGCCGTATTCCTCTGG |
| *Dβ3 (IsoformA)* | *CG11822* | ACGAACTACGACAGCGATGTGCAG | TCCTCGTCTCTCCATCGGAGATTC |
| *rp49* | *CG7939* | AGCTGTCGCACAAATGGCGCAAGC | TTGAATCCGGTGGGCAGCATGTGG |
